# Early-life microbiota in the LIMIT cohort: unveiling meconium microbiota types beyond maternal lifestyle

**DOI:** 10.1080/19490976.2026.2712811

**Published:** 2026-08-04

**Authors:** Dana El Masri, Niccolò Meriggi, Federica Loperfido, Irene Bianco, Chiara Ferrara, Beatrice Maccarini, Francesca Sottotetti, Carlotta De Filippo, Nicolò Loddo, Rosa Maria Cerbo, Stefano Ghirardello, Francesca Garofoli, Micol Angelini, Maria Cristina Monti, Mulubirhan Assefa Alemayohu, Mie Bech Lukassen, Hellas Cena, Rachele De Giuseppe

**Affiliations:** a Laboratory of Dietetics and Clinical Nutrition, Department of Public Health, Experimental and Forensic Medicine, University of Pavia, Pavia, Italy; b Institute of Agricultural Biology and Biotechnology (IBBA), National Research Council (CNR), Pisa, Italy; c Neonatal Unit and Neonatal Intensive Care Unit, Fondazione IRCCS Policlinico San Matteo, Pavia, Italy; d Biostatistics and Clinical Epidemiology Unit, Department of Public Health, Experimental and Forensic Medicine, University of Pavia, Pavia, Italy; e DNASense (Cmbio), Aalborg Øst, Denmark; f Clinical Nutrition and Dietetics Service, Unit of Internal Medicine and Endocrinology, ICS Maugeri IRCCS, Pavia, Italy

**Keywords:** Early gut microbiota, meconium analysis, gestational weight gain, pre-pregnancy BMI, 16S rRNA sequencing, unsupervised analysis, lifestyle factors

## Abstract

**Introduction:**

Early gut microbiota development plays an important role in lifelong human health, and meconium offers a unique matrix to understand prenatal microbial exposures. This study from the LIMIT cohort aimed at investigating associations between maternal pre-pregnancy body mass index (pre-BMI) and gestational weight gain (GWG), as well as other maternal lifestyle and environmental factors, with the structure of meconium microbiota at delivery.

**Methods:**

Two hundred pregnant women were enrolled during the pre-hospital care before birth at Fondazione IRCCS Policlinico San Matteo (Pavia), according to the inclusion/exclusion criteria. Mothers were grouped based on their GWG gain category according to the IOM guidelines. 168 meconium samples were analyzed using a targeted long-reads sequence approach. Bacterial 16S rRNA (V1–V8) amplicons were sequenced using the Oxford Nanopore PromethION platform. Supervised analyses assessed associations between maternal variables and bacterial diversity, while an unsupervised learning approach based on the Partitioning Around Medoids (PAM) clustering algorithm was applied to identify specific microbial clusters unrelated to the cohort's metadata.

**Results:**

No significant associations were observed between GWG, pre-BMI or other lifestyle factors and overall microbial diversity. Unsupervised learning revealed five distinct meconium microbial clusters, i.e. “microbiota-types”, related to different taxonomic profiles, determined by the variations in specific bacterial species.

**Conclusion:**

No significant influence of the studied variables was observed on meconium microbiota. However, the meconium microbial communities appear to organize into enterotype-like structures, highlighting the need for longitudinal studies to identify key determinants of early colonization.

## Introduction

The establishment of the gut microbiota during the early stages of life is a crucial process for maintaining overall human health.^1^ Initial microbial colonization of the neonatal gastrointestinal tract plays a fundamental role in the development of the immune system, metabolism, and brain function.^1^ ^,^ ^2^ Any disruption in this process may contribute to adverse short- and long-term health consequences, including necrotizing enterocolitis (NEC), obesity, asthma, diabetes, inflammatory bowel disease, cancer, allergies, and neurological conditions related to the gut-brain axis.^1^

Over the past decade, some studies have suggested the potential presence of microbial communities in meconium and fetal tissues, supporting the hypothesis of in utero microbial exposure.^3^ However, these findings remain limited, methodologically challenging, and subject to ongoing debate.^4^ Current evidence indicates that, in the vast majority of healthy pregnancies, the neonatal gut microbiota is established primarily at birth, with maternal microbes acquired during delivery, as well as through early maternal-infant contact and initial environmental exposures representing the principal sources of early colonization.^4^

Nevertheless, maternal factors during pregnancy may influence fetal microbial and immune development indirectly, through multiple interconnected pathways, including the transplacental transfer of microbial metabolites, exposure via the umbilical cord or amniotic fluid, fetal membranes, and gastrointestinal tract.^1^ Evidence indicates that microbiome-related metabolites originating from the maternal microbiota are transferred vertically to the fetus. Short-chain fatty acids (SCFAs) and secondary bile acids appear to play active roles in modulating the fetal immune system and preparing the gut for postnatal microbial exposure.^5^

Beyond prenatal microbial exposure, a range of maternal and environmental factors could be implicated in shaping early-life microbiota development during the first 1000 d. These include pre-BMI,^6^ GWG,^6^ maternal dietary habits,^7^ delivery mode,^8^ infant feeding practices,^8^ antibiotic usage,^8^ and various environmental exposures.^8^ Several studies reported that maternal lifestyle factors, along with weight status, have been associated with variations in newborn gut microbial diversity and abundance.^6^ ^,^ ^7^ ^,^ ^9^

An observational study of 935 mother-infant pairs found that infants born vaginally to mothers with overweight or obesity before pregnancy, had higher abundance of Firmicutes, especially the Lachnospiraceae family, and were three times more likely to develop overweight or obesity (95% CI, 1.49–7.41) by ages 1 and 3 y old, when compared to those born to mothers with normal pre-BMI.^10^ This risk increased further among infants delivered by caesarean section to overweight mothers, who were five times more likely to become overweight (95% CI, 2.04–12.38) at the same ages.^10^ Similarly, a study by Mueller NT et al.^11^ involving 74 neonates to mothers with a pre-BMI indicating overweight or obesity, revealed changes in infants' microbiota, particularly among those born via vaginal delivery.^11^ Another study conducted by Sasaki T. et al.^7^ on 33 mother–infant pairs demonstrated that higher maternal adherence to a Mediterranean Diet (MD) during pregnancy is associated with increased abundance of *Pasteurellaceae*, *Bacteroidaceae*, and other SCFAs-producing bacteria in neonatal meconium.^7^ In addition to microbiota changes, the study^7^ also found an association with epigenetic differences in placental and cord blood tissues, suggesting that maternal diet can influence early gut microbiota development not only through the direct transfer of microbes, but also by altering fetal gene activity via influences on the gut and immune system function.^7^

Since meconium begins to develop in the fetal intestine during the late stages of gestation, it represents a valuable matrix for investigating early microbial and metabolic exposures. Therefore, analyzing meconium provides a unique opportunity to better understand the prenatal microbial environment and its potential implications for the subsequent development of the gut microbiota during the first 1000 d of life.^12^ Despite such findings, the influence of maternal factors during pregnancy on the composition of meconium microbiota is still lacking and needs further investigation.

In this context, using data from the LIMIT (Lifestyle and Microbiome Interaction Early Adiposity Rebound in Children) cohort study,^13^ we investigated whether maternal pre-BMI, GWG adequacy (classified as adequate or inadequate according to the Institute of Medicine guidelines^14^) and maternal lifestyle factors during pregnancy (e.g., dietary habits, physical activity level, smoking habits) were associated with the meconium microbiota structure. While these predefined maternal variables showed no effect in explaining the observed biological variability within the samples, the application of unsupervised learning approaches enabled the identification of hidden patterns of bacterial variation. Specifically, by applying a Partitioning Around Medoids (PAM) algorithm, we uncovered distinct and biologically meaningful bacterial community structures that were not captured by traditional hypothesis-driven analyses. These latent clusters reflected intrinsic biological heterogeneity within the meconium microbiota, highlighting the capacity of unsupervised learning-based methods to reveal hidden structure in complex, low-biomass substrates and underscores the importance of these approaches in microbiome research, particularly when investigating early-life samples.

## Materials and methods

### Study cohort description

The present research refers to the experimental hypothesis of the ongoing prospective cohort study LIMIT (www.clinicaltrials.gov, NCT04960670)^13^ which investigates the longitudinal interplay between infant gut microbiome, infant/maternal lifestyle exposure factors, and environmental variables at different follow-up (T_0_, at delivery; T_1_, 1 month; T_2_, 6 months; T_3_, 12 months; T_4_, 24 months; T_5_, 36 months after birth).

Ethical approval was granted by the Ethical Committee of IRCCS Policlinico San Matteo (Pavia) (protocol number: 0020200/22; Accepted: 11 April 2022).

A total of 200 pregnant women were enrolled between October 2022 and June 2024 during the pre-hospital care before birth at the UOC Neonatology and Neonatal Intensive Care, Fondazione IRCCS Policlinico San Matteo of Pavia (Pavia, Italy). The overall selection was performed based on the inclusion and exclusion criteria previously defined in the LIMIT study protocol.^13^ Additionally, the completeness of the data and the availability of a neonatal meconium sample at T_0_ for gut microbiota analysis were added as additional inclusion and exclusion criteria. Therefore, 168 mother-infant pairs were selected for this specific study.

The study adhered to the principles outlined in the Helsinki Declaration, and all participants signed a consent form. In the current analysis, we analyzed meconium samples collected at birth from newborns. At the delivery, women were investigated for potential modifiers of the microbial composition, including: (i) sociodemographic characteristics (such as age and educational level); (ii) pre-pregnancy anthropometric data (pre-BMI) and GWG adequacy categories^14^; (iii) lifestyle factors during pregnancy, including dietary habits, physical activity level, and smoking habits, were collected through the LIMIT study^13^ by using previously validated questionnaires and structured interviews.

### Maternal anthropometric measurements

Maternal pre-pregnancy weight and height were self-reported by the participants. BMI was calculated using weight (Kg) divided by height squared (m^2^) and used to classify participants as underweight (BMI < 18.5 kg/m^2^), normal weight (18.5 ≤ BMI ≤ 24.9 kg/m^2^), overweight (25 ≤ BMI ≥ 29.9 kg/m^2^) or with obesity (BMI ≥ 30 kg/m^2^).^15^ The total weight gained during pregnancy was also self-reported. Total GWG was calculated as the difference between pre-pregnancy weight and total weight at delivery. According to the IOM guidelines,^14^ participants were classified into (i) adequate GWG (AGWG): women who gained within the recommended range for their respective pre-BMI category; (ii) excessive GWG (EGWG): women who gained more than the recommended range for their respective pre-BMI category; (iii) low GWG (LGWG): women who gained less than the recommended range for their respective pre-BMI category.

### Lifestyle variables assessment

Detailed descriptions of the questionnaires, scoring systems and interviews for lifestyle data collection are provided in the previous publication of the LIMIT study protocol.^13^

In brief, Food consumption frequency (FFQ) and dietary habits (DH) were assessed using a previously validated self-administered dietary questionnaire.^16^ Two of the nine original sections, initially developed for an Italian adolescent population,^16^ were adapted to adults by two dietitians, as described elsewhere.^17^ The FFQ section (18 items) evaluated daily consumption of staple foods and beverages (e.g., bread, pasta, cereals, fruits, vegetables, milk, yoghurt, tea, coffee), and weekly intake of foods such as meat, fish, eggs, cheese, legumes, sweets, and alcohol.^16^ Responses were based on a four-point Likert scale (always, often, sometimes, never), scored from 0 to 3, with higher scores reflecting healthier choices.^16^ The DH section (14 items) investigated dietary habits, including breakfast frequency, number of daily meals, fruit and vegetable intake, and beverage consumption.^16^ Eight items use the same four-point scale, while six have alternative formats scored from 1 to 4. In both cases, the highest score indicates the healthiest behavior.^16^ Total scores were divided into tertiles: “inadequate,” “partially satisfactory,” and “satisfactory” dietary habits, aligned with the Italian National Dietary Guidelines.^18^

The adherence to the MD was evaluated through the MEDI-LITE score, a validated index that measures dietary patterns based on the frequency of consumption of nine key food groups characteristic of the MD, such as vegetables, fruits, legumes, cereals, and olive oil.^19^ For this study, the scoring system was adapted to suit the specific nutritional needs and dietary habits of pregnant women.^20^ Therefore, after excluding the alcohol component, the total score ranged from 0 to 16, with higher values indicating greater adherence to the MD. Scores were categorized into tertiles representing low, medium, and high levels of adherence.

Physical activity (PA) levels during pregnancy were assessed using a section of a previously developed and validated questionnaire for the Italian youth population,^16^ which was adapted for use with adults by removing items related to school-based physical activities. The modified version was pre-tested on a sample of 24 individuals and subsequently revised based on the feedback received.^20^ The questionnaire aimed to assess PA patterns by quantifying the time spent weekly on various activities, including leisure-time physical activity and screen time.^16^ It consisted of five multiple-choice items, each presented in a 4-point Likert scale format (always, often, sometimes, never), with responses scored from 0 to 3. Higher scores indicated healthier lifestyle behaviors; scores were then categorized into tertiles representing low, medium, and high levels of PA.

Smoking habits were assessed through interviews, and participants were asked to report the number of cigarette packs smoked per year. Based on their responses, women were categorized as either smokers during pregnancy (“yes”) or non-smokers during pregnancy (“no”).

### Meconium sample collection and DNA extraction

Meconium samples were collected within the first 24 h of birth using sterile protocols and stored at −80 °C until analysis, as recommended by previous studies.^3^ ^,^ ^21^ The fecal samples were shipped on dry ice and sequenced. DNA extraction was carried out using a revised DNeasy 96 PowerSoil Pro QIAcube HT Kit method as described by Jensen et al.^22^ Modifications include mixing each sample with 500 µL of CD1 solution, followed by bead-beating at 1600 rpm for two minutes, repeated three times, with two-minute breaks on ice in between. After lysis, samples were spun at 3500 × *g* for ten minutes. The supernatant of each sample was transferred into a new S-block well and mixed with CD2 and nuclease-free water in the ratio 3:3:1 (V/V). Contents were thoroughly mixed, and samples were then spun again at 3500 ×* g* for 10 min.

### Bacteria 16S rRNA gene variable regions 1–8 (bV18-A) library preparation and sequencing

Amplicon libraries targeting the bacterial 16S rRNA gene variable regions 1–8 (bV18-A) were generated following a customized protocol. For each reaction, up to 25 ng of purified DNA served as the template for PCR amplification. The 50 μL PCR mix included 0.2 mM of each dNTP, 0.01 units of Platinum SuperFi DNA Polymerase (Thermo Fisher Scientific, USA), and 500 nM of both forward and reverse primers, prepared in the SuperFi Buffer provided by the manufacturer. PCR was done with the following program: Initial denaturation at 98 °C for 3 min, 25 cycles of amplification (98 °C for 30 s, 62 °C for 20 s, 72 °C for 2 min) and a final elongation at 72 °C for 5 min. Both forward and reverse primers include custom 24 nt barcode sequences followed by the sequences targeting bV18-A: [8F] AGRGTTYGATYMTGGCTCAG and [1391R] GACGGGCGGTGWGTRCA.^23-26^ The obtained amplicon libraries were purified following the standard CleanNGS SPRI bead protocol (CleanNA, NL) using a bead-to-sample ratio of 3:5. Purified DNA was eluted in 25 μL of nuclease-free water (Qiagen, Germany). Sequencing-ready libraries were then constructed from these purified amplicons with the SQK-LSK114 kit (Oxford Nanopore Technologies, UK), following the manufacturer's guidelines with minor adjustments: a total of 500 ng DNA was used as input, and CleanNGS SPRI beads were employed during all clean-up steps. DNA concentration was quantified using the Qubit dsDNA HS Assay Kit (Thermo Fisher Scientific, USA). To assess fragment size and purity, a subset of amplicon libraries was analyzed by gel electrophoresis on a Tapestation 2200 using D1000 or High Sensitivity D1000 Screentapes (Agilent, USA). The final sequencing library was loaded onto a PromethION R10.4.1 flow cell and sequenced using the MinKNOW 24.06.15 software (Oxford Nanopore Technologies, UK). Reads were basecalled and demultiplexed with MinKNOW Dorado 7.4.14 using the super accurate basecalling algorithm (config r10.4.1_400bps_sup.cfg) and custom barcodes.

### Statistical analysis of maternal sociodemographic and lifestyle factors

Descriptive statistics were used to summarize the sociodemographic, anthropometric, and lifestyle characteristics of the study population. Continuous variables were reported as means and age range, while categorical variables were expressed as frequencies and percentages. Differences across GWG categories, classified as LGWG, AGWG, or EGWG, were assessed using the Kruskal–Wallis test for continuous variables and the Chi-square test for categorical variables. Since the sample related to each mother considered in the present analysis was substantially similar to that examined in our previous study,^20^ differing only by a few mother-infant pairs based on the availability of meconium samples at T0, the same multinomial logistic regression model was applied to identify factors associated with GWG categories, using AGWG as the reference category. This approach was adopted to confirm the consistency of the previously reported findings.

The results were expressed as relative risk ratios (RRRs) with corresponding 95% confidence intervals (CIs). All statistical tests were two-sided, and a *p*-value < 0.05 was considered indicative of statistical significance. Analyses were performed using StataCorp. 2024. Stata Statistical Software: Release 18.5. College Station, TX: StataCorp LLC.

### Long-read bioinformatic processing and statistical analyses

Sequencing reads obtained from the demultiplexed and basecalled FASTQ files were subjected to length (320–2000 bp) and quality (phred score > 17) using a local implementation of filtlong v0.2.1 with the settings–min_length 320–max_length 2000–min_mean_q 98. The resulting high-quality reads were then aligned to the representative (reps) 16S rRNA (SSU) sequences from the GTDB release 220 reference database with minimap2 v2.24-r1122 using the -ax map-ont command,^27^ followed by downstream processing with samtools v1.14.^28^ The combined dataset, containing both bacterial and archaeal sequences (bac120_ssu_reps_r220.fna and ar53_ssu_reps_r220.fna) was further refined by retaining archaeal SSU > 450 bp, and bacteria SSU > 650 bp. Mapping results were filtered to retain only alignments where the query-to-alignment length ratio deviated by less than 5%. Mapped GTDB representative genome identifiers were referred to as Operational Taxonomic Units (OTUs). Notably, low–low-abundance OTUs contributing less than 0.1% of the total mapped reads within per sample were excluded as part of a data-cleaning step.

All statistical analyses were performed in R Software v4.3.1.^29^ The OTUs sequences were first screened for non-standard nucleotide symbols, and any invalid characters were removed prior to further processing. The cleaned sequences, along with the OTU count table and metadata, were imported into an ampvis2 object using the “ampvis2” package v2.8.9.^30^ To prevent confounding effects and spurious associations, samples related to c-section cases were excluded from the dataset. β-diversity analyses were conducted by using the “vegan” package v2.6.10.^31^ and differences in β-diversity were depicted in multidimensional space using Principal Coordinates Analysis (PCoA) generated with the *cmdscale* function on the Bray–Curtis dissimilarity matrix. α-diversity analysis was computed using the *amp_alphadiv* function of the “ampvis2” package v2.8.9. Environmental fitting tests were carried out by using the *envfit* function of the “vegan” package v2.6.10. Latent structure in the data was identified using unsupervised clustering with the Partitioning Around Medoids (PAM) algorithm, implemented in the *pam* function of the “cluster” package v2.1.4.^32^ Original OTUs-related fasta files containing the primary OTU identifier together with the OTUs abundance table were used to run PICRUSt2 v2.6.3 pipeline.^33^

Multivariable associations between predicted bacterial functions and host clinical variables were assessed using MaAsLin2 v1.16.0.^34^ Independent linear models were fitted for each KEGG Ortholog (KO), Enzyme Commission (EC) number, and MetaCyc pathway and OTUs abundance, including gestational age, gestational weight gain (GWG), maternal age and pre-BMI as fixed effects, with adequate GWG as the reference category. Because PICRUSt2 outputs represent copy number-corrected predicted functional abundances, no additional normalization was applied to KO, EC, and MetaCyc pathway datasets. Differently, the OTUs abundances were normalized using total sum scaling (TSS). All features detected in fewer than 5% of samples were excluded prior to analysis for each dataset, and the remaining abundances were log-transformed using the default MaAsLin2 transformation method before multivariable modelling. All the methodological details concerning the statistical analyses performed were reported in detail in the supplementary information file.

## Results

### Maternal sociodemographic and lifestyle factors

Table 1 presents the distribution of sociodemographic and lifestyle factors of the study participants according to the GWG category.^14^ Overall, 58.34% of women in the sample were classified as having inadequate GWG, of whom 68.37% were in the LGWG category and 31.64% in the EGWG category. The median age of participants was similar across GWG groups: 33 y (IQR 31–36) for AGWG, 32 y (IQR 29–36) for EGWG, and 33 y (IQR 30–36) for LGWG, with no statistically significant differences observed. Similarly, the educational level did not differ significantly among the groups. For the pre-BMI variable, a statistical significance was observed (*p* < 0.001); notably, 45% of the participants in the EGWG category (*n* = 31) had a pre-BMI indicating overweight (35%, *n* = 11) or obesity (10%, *n* = 3) (Table 1).

Concerning gestational lifestyle habits, no significant associations were observed between GWG categories and adherence to the MD. However, only 43% of women in the AGWG group reported medium or high adherence to the MD, compared to 55% and 52% in the LGWG and EGWG groups, respectively. Conversely, low adherence to the MD was more prevalent in the EGWG group, encompassing 72% of its participants (Table 1).

Concerning the FFQ score, a trend was observed (*p* = 0.082), suggesting an association between GWG categories and food frequency consumption. Indeed, among women with EGWG, 35% showed inadequate scores, 52% partially satisfactory, and only 13% satisfactory. In the AGWG group, 40% had inadequate scores, 39% partially satisfactory, and 21% satisfactory. Conversely, among those with LGWG, a higher proportion (36%) achieved satisfactory scores, while 39% were partially satisfactory and 25% inadequate. A similar trend was observed for the DH score (*p* = 0.068), where 48% of women in the EGWG group had inadequate scores, compared to 43% in the AGWG group and 30% in the LGWG group. The proportion of satisfactory scores was highest among women with LGWG (31%), followed by those with EGWG (29%) and AGWG (16%).

PA levels also showed no significant differences across the GWG categories. Nevertheless, medium or high PA scores were reported by 70% of participants in the AGWG group and 69% in the LGWG group (Table 1). Finally, a statistically significant difference was found in smoking habits during pregnancy (*p* < 0.001). 13% of women in the EGWG group reported smoking while pregnant (Table 1).

**Table 1.** Maternal sociodemographic and lifestyle factors related to gestational weight gain.

	EGWG	AGWG	LGWG	*p*-value
***n***	31	70	67	
**Age (year)**	32.00 (29.00–36.00)	33.00 (31.00–36.00)	33.00 (30.00–36.00)	0.20
**Mother's educational Level**				0.061
Lower or high school	6 (20%)	7 (10%)	3 (5%)	
University degree	24 (80%)	63 (90%)	63 (95%)	
**Pre-pregnancy BMI category**				**<0.001**
Underweight (BMI < 18.5 kg/m^2^)	0 (0%)	7 (10%)	9 (13%)	
Normal weight (18.5 ≤ BMI ≤ 24.9 kg/m^2^)	17 (55%)	54 (77%)	53 (79%)	
Overweight (25 ≤ BMI ≥ 29.9 kg/m^2^)	11 (35%)	5 (7%)	3 (4%)	
Obesity (BMI ≥ 30 kg/m^2^)	3 (10%)	4 (6%)	2 (3%)	
**Food frequencies (FFQ)**				0.082
Inadequate	11 (35%)	28 (40%)	17 (25%)	
Partially satisfactory	16 (52%)	27 (39%)	26 (39%)	
Satisfactory	4 (13%)	15 (21%)	24 (36%)	
**Dietary habits (DH)**				0.068
Inadequate	15 (48%)	30 (43%)	20 (30%)	
Partially satisfactory	7 (23%)	29 (41%)	26 (39%)	
Satisfactory	9 (29%)	11 (16%)	21 (31%)	
**Mediterranean diet adherence score category [without alcohol]**				0.50
Lower score	15 (48%)	40 (57%)	30 (45%)	
Medium score	7 (23%)	18 (26%)	19 (28%)	
Higher score	9 (29%)	12 (17%)	18 (27%)	
**Physical activity**				0.45
Lower score	15 (48%)	21 (30%)	21 (31%)	
Medium score	8 (26%)	28 (40%)	24 (36%)	
Higher score	8 (26%)	21 (30%)	22 (33%)	
**Did you smoke during pregnancy?**				**<0.001**
No	16 (52%)	24 (34%)	12 (18%)	
Yes	4 (13%)	3 (4%)	0 (0%)	

Notably, although FFQ and DH were initially assessed using two validated sections of the Turconi et al. questionnaire,^16^ these variables were not analyzed separately nor included in the final multivariable analyses. Instead, we focused exclusively on the MEDI-LITE score as a measure of adherence to the MD. This decision was based on both methodological and conceptual considerations.

First, the MEDI-LITE score is a comprehensive, validated index specifically designed to assess adherence to the MD pattern, which was the primary dietary framework of interest in our study. Unlike the FFQ and DH sections, which provide more general insights into dietary behavior and consumption frequency, the MEDI-LITE score directly reflects alignment with a well-characterized and evidence-based dietary model known to influence gut microbiota composition.^35^ Second, including all three scores in the multivariate model would have introduced redundancy, as several components of the Turconi-derived scores overlap conceptually with the MEDI-LITE components (e.g., fruit and vegetable intake, frequency of sweets or meat consumption). This collinearity could have affected the robustness and interpretability of the regression models, resulting in misleading results.

No significant differences were observed in daily fruit consumption across GWG groups; most women reported average fruit intake, with proportions ranging from 55% in the EGWG group to 68% in the LGWG group. In contrast, a statistically significant difference emerged for daily vegetable consumption (*p* = 0.031); women in the AGWG group most frequently reported average vegetable intake (86%), whereas EGWG and LGWG groups showed a higher prevalence of both inadequate (18% and 15%, respectively) and adequate (21% and 18%, respectively) consumption levels. Weekly legume consumption did not differ significantly among groups, although a higher proportion of women in the AGWG and LGWG categories reported average intake (55% and 58%, respectively), compared to only 37% in the EGWG group. Daily cereal consumption and weekly fish consumption were not significantly associated with GWG, and across all groups, with average intake reported by most participants in all groups. Daily meat consumption was evenly distributed among the three groups, with most women in each category consuming between 1 and 1.5 portions per day or less than one portion. Although not reaching statistical significance (*p* = 0.054), differences in daily milk and dairy product consumption approached significance and women with AGWG most frequently consumed 1 to 1.5 portions per day (60%), whereas both LGWG and EGWG groups had a higher proportion of women reporting consumption below or above this range. Lastly, regular use of olive oil was the most common across all GWG categories, particularly among women with AGWG (71%), showing no significant differences among the categories.

**Figure 1.**
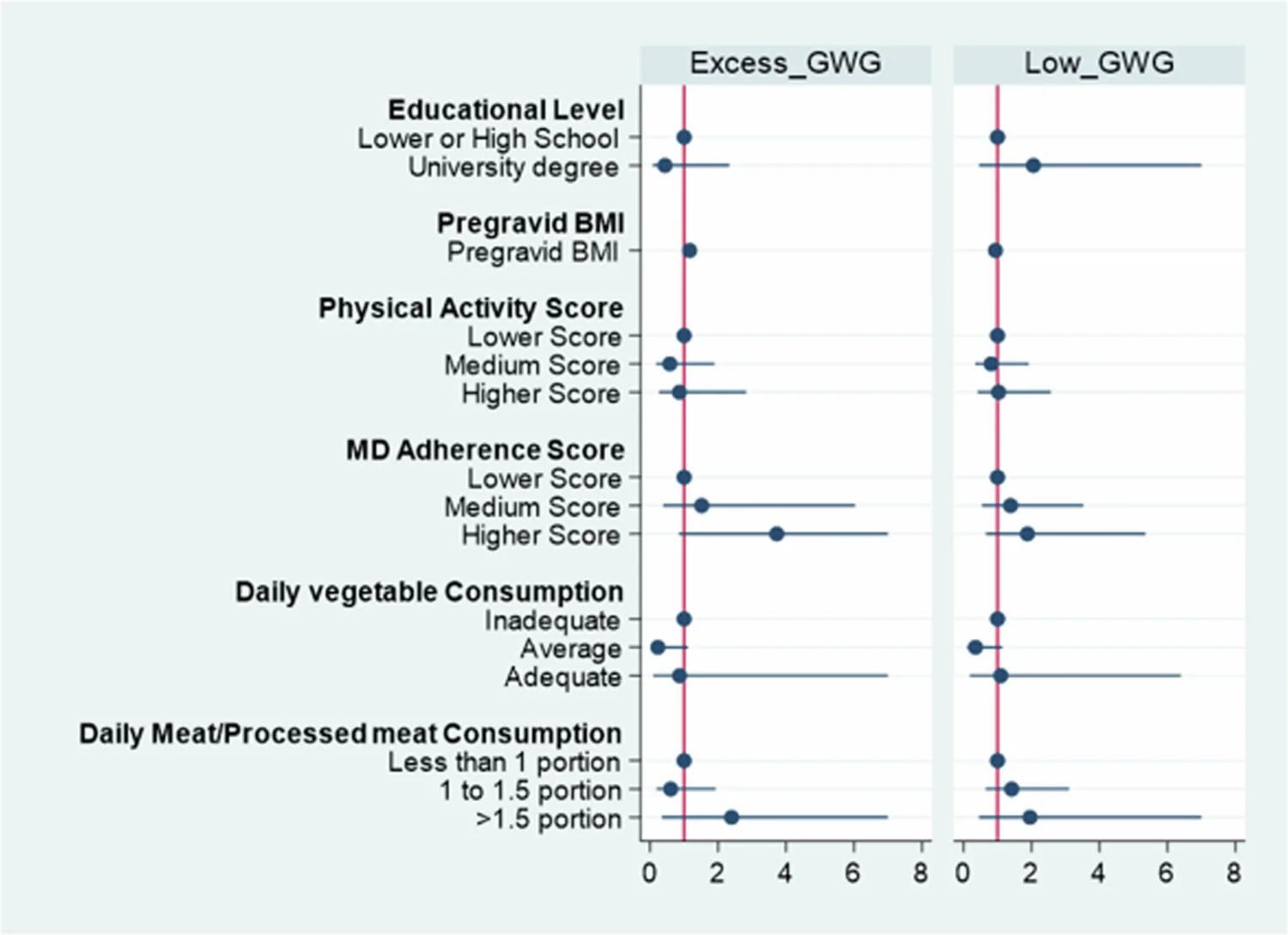
The forest plot illustrates the results of the multinomial regression model based on maternal data from the LIMIT prospective cohort study. RRRs and corresponding confidence intervals for EGWG and LGWG are presented, using AGWG as the reference category.

A multinomial regression analysis was conducted to identify dietary, anthropometric and social demographic predictors associated with GWG categories, using AGWG as the reference group. The results are presented in Figure 1.

Pre-BMI was significantly associated with an increased risk of EGWG compared to AGWG (Relative Risk Ratio [RRR] = 1.162, *p* = 0.007). Additionally, a trend toward a protective effect was observed for daily vegetable consumption: women reporting an average intake of 1 to 1.5 portions per day had a lower risk of EGWG compared to those with AGWG (RRR = 0.227, *p* = 0.068), although this association did not reach conventional statistical significance.

No other variables included in the model were significantly associated with LGWG relative to the reference category.

### Meconium microbiota development according to maternal dietary parameters

**Figure 2.**
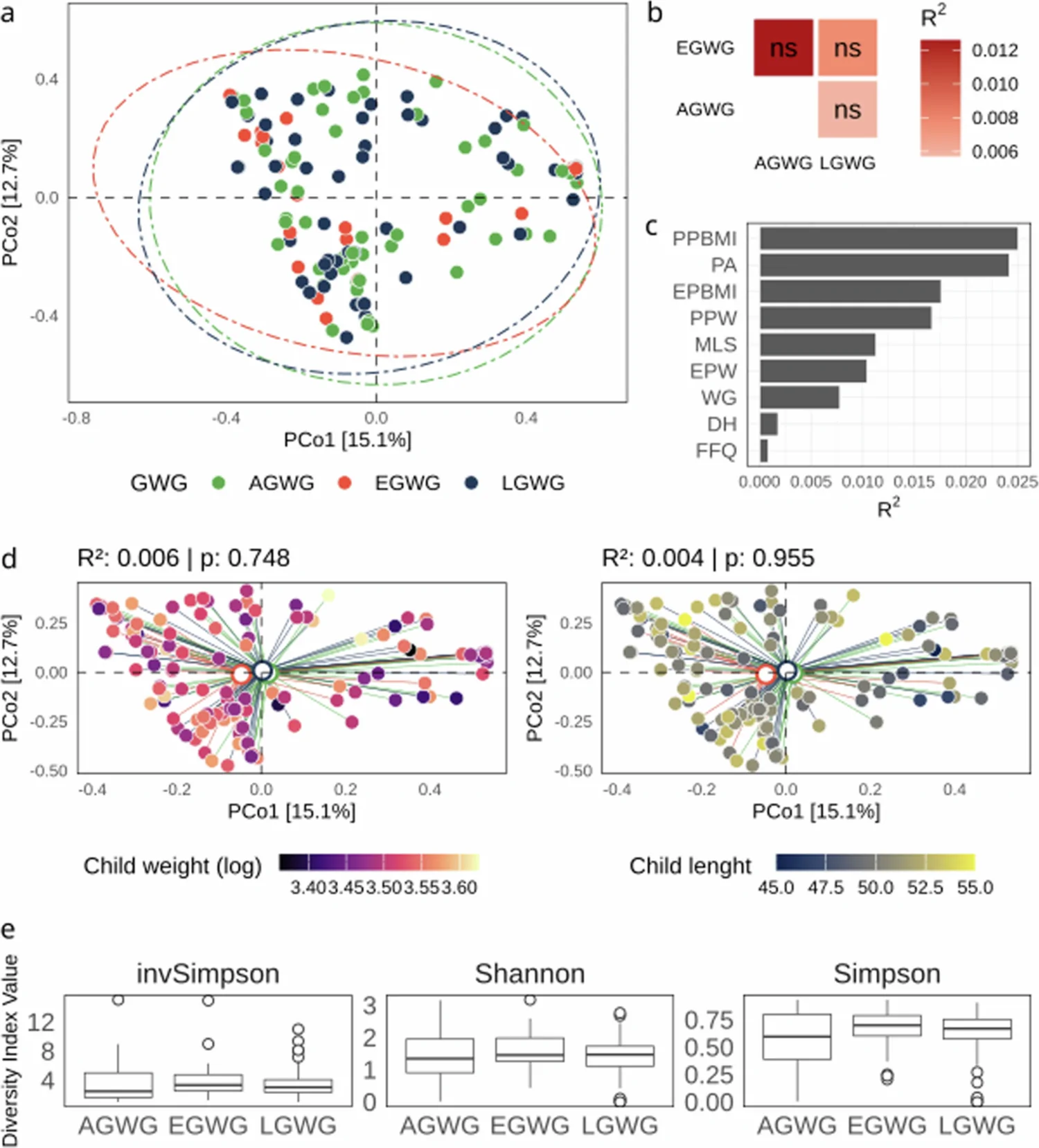
Effect of GWG on meconium bacterial diversity in infants born via vaginal delivery. (a) PCoA (Bray–Curtis distance) based on the relative abundance table depicted according to GWG by using a color scheme. The first two principal coordinate axes (PCo1 and PCo2), which explained 15.1% and 12.7% of the total variation, respectively, were used to highlight a sample distribution. The 95% confidence ellipses were added using the dotted line. (b) Heatmaps report the results of pairwise Adonis PERMANOVA comparisons between GWG groups. The significance is annotated within each tile using annotation (ns: non-significant results). Each tile represented a pairwise comparison, colored by the corresponding R^2^ value. (c) Barplot reports the amount of variance (i.e. R^2^ from the *envfit* function) for each maternal anthropometric and lifestyle variable tested in the model formula. All variables tested display no significant effect (*p* > 0.05). (d) PCoA/spider plot ordination (Bray–Curtis distance on relative abundance counts) display the distribution of samples by GWG represented using centroids (empty circles according to GWG color scheme) and the child anthropometric characteristics (weight and length) reported using color gradient and tested using adonis PERMANOVA analysis. The adonis PERMANOVA outcomes (R^2^ and *p*-value) are reported on top of each panel. (e) Boxplot reports the α-diversity metrics, Inverse Simpson, Shannon, and Simpson indices among different GWG categories. Differences between α-diversity metrics are assessed using the Kruskal–Wallis test. Abbreviations: PPBMI, pre-pregnancy body mass index; WG, weight gain; PPW, pre-pregnancy weight; EPW, end-pregnancy weight; EPBMI, end-pregnancy body mass index; FFQ, food frequency questionnaire score; DH, dietary habits; PA, physical activity; MLS, MEDI-LITE score.

**Figure 3.**
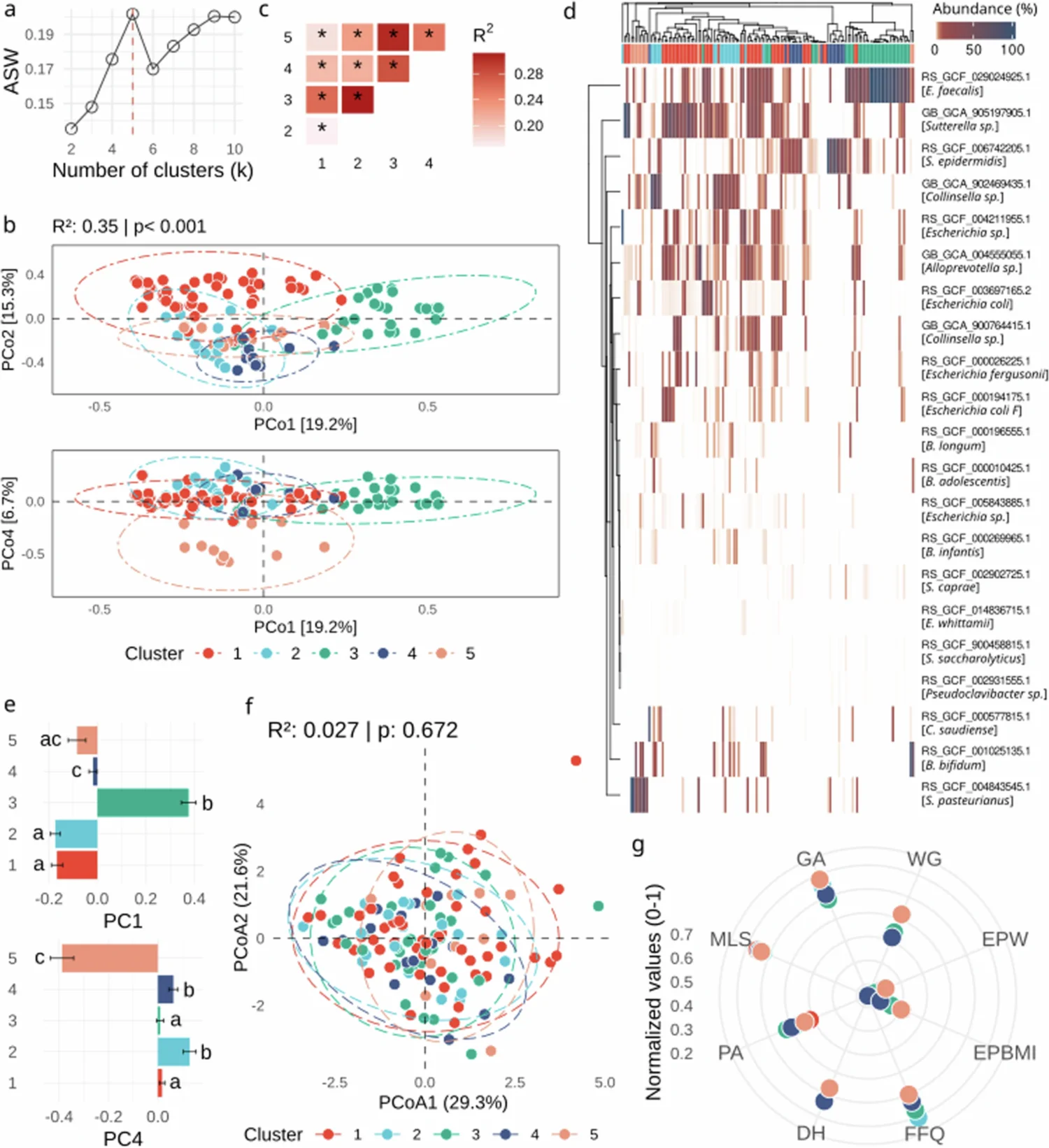
Assessment of different microbiota types through unsupervised learning analysis in infants born via vaginal delivery. (a) The line chart reports the Average Silhouette Width (ASW) calculated for the interval between 2 and 10 clusters. Each point represents the ASW obtained for each cluster, and the vertical red dotted line highlights the optimum number of clusters. (b) PCoA based on Bray–Curtis distance, produced using the axis PCo1 vs PCo2 and PCo1 vs PCo4, highlights the sample distribution according to the clusters calculated by using the PAM algorithm. Clusters are represented using color code in the legend, and ellipses represent 95% confidence intervals of the clusters. (c) The heatmap represents the results of cluster cross-comparisons produced by pairwise adonis PERMANOVA. Significant contrasts are reported using asterisks (*p < 0.001) while the R^2^ values are reported using a color gradient. (d) Heatmap reports the distribution of significantly different OTUs across clusters selected using the Wilcoxon test (FDR adjustment). The OTUs are reported in the rows, and each column corresponds to a sample. Percentage abundances are shown on a color scale ranging from low (white) to high (dark red-blue). Top annotations indicate cluster membership, with colors assigned according to the color scheme in the legend. Rows were hierarchically clustered using Euclidean distance and the Ward D2 linkage method. Each OTU label is reported together with the related taxonomic assignment, indicated below each OTU using brackets. (e) Barplot reports the cluster-wise mean values and standard errors of first (PC1) and fourth (PC4) axis for each cluster (color code). Letters above bars indicate statistically distinct groups based on Dunn's post-hoc tests (FDR correction). Clusters sharing the same letter are not significantly different from each other, whereas clusters with different letters are significantly different. (f) Principal Coordinates Analysis (PCoA) based on Euclidean distances computed from z-score-standardized maternal dietary and lifestyle variables (GA, WG, EPW, EPBMI, FFQ, DH, PA, and MLS). Each point represents an individual subject, colored by cluster, with 95% confidence ellipses shown for each group. Axis labels indicate the percentage of variance explained. Adonis PERMANOVA results are reported on top of the panel (g) Radar plot showing cluster-wise mean normalized values (min-max scaling, 0–1) for the maternal dietary and lifestyle variables (WG, EPW, EPBMI, FFQ, DH, PA, and MLS). Abbreviations: GA: Gestational age (weeks); WG, weight gain (gestational weight gain in Kg); EPW, end-pregnancy weight; EPBMI, end-pregnancy body mass index; FFQ, food frequency section; DH, dietary habits; PA, physical activity; MLS, MEDI-LITE score.

The ordination analysis (PCoA based on Bray–Curtis distance; Figure 2a) did not reveal any evident grouping of samples according to GWG categories. This observation was consistent with the results of the permutational multivariate analysis of variance (PERMANOVA; Supplementary Table S1), which showed no significant effect of GWG on bacterial diversity, corroborated by comparing all group combinations by using pairwise adonis PERMANOVA (Figure 2b and Supplementary Table S3). Therefore, there were no significant differences in β-diversity among meconium samples from mothers with AGWG, EGWG, or LGWG. A lack of significant effect on bacterial diversity was also observed for all main variables included in the model formula (gender, pre-BMI, GWG, MEDI-LITE score, MEDI-LITE score rank, celiac condition, PA, FFQ, and IPAQ), as assessed by adonis PERMANOVA (Supplementary Table S1). Environmental fitting analysis was also conducted to examine associations between main study variables (continuous variables) and bacterial community structure. Similarly, no environmental or lifestyle variable was found to significantly correlate with the primary axes of variation (Figure 3c and Supplementary Table S2), suggesting that the observed sample distribution may reflect complex, multivariate factors not directly related to the studied variables. Maternal age, gestational age, and neonatal anthropometric measures were also evaluated. Adonis PERMANOVA analyses showed no significant associations between these variables and meconium microbial diversity (*R* ^2^ = 0.004–0.007; all *p* > 0.59). Consistently, PCoA based on infant weight and length showed substantial overlap among samples, with their distribution visualized relative to GWG group centroids, indicating no distinct clustering even according to GWG categories (Figure 2d). Furthermore, no significant differences in α-diversity were observed across GWG categories (adequate, excessive, and low) (Figure 2e).

### Unsupervised learning reveals distinct microbiota-type patterns

As no significant associations were observed between bacterial diversity and the study-defined categories, we performed a deeper exploration of the dataset by performing an unsupervised learning analysis aimed at identifying coherent clusters independently of our study categories.

To identify natural groupings within the sample dataset, a PAM clustering approach was applied. The optimal number of clusters (k) was determined using average silhouette width (ASW), which peaked at an optimum of 5 clusters, indicating the most appropriate partitioning structure (Figure 3a). Cluster 1 represented the most prevalent community type, accounting for 37.4% of the samples, followed by Cluster 3 (26.0%), whereas Cluster 5 was the least represented within the samples dataset (9.2%) (Supplementary Table S4). The unsupervised classification resulted in 5 distinct clusters reported by using ordination analysis (PCoA, Bray–Curtis distance in Figure 3b), depicted different principal coordinate axes to highlight cluster separation better. Permutational multivariate analysis adonis PERMANOVA confirmed that the identified clusters accounted for a significant portion of the variation in microbial community composition (R^2^ = 0.35, *p* = 0.001), highlighting that the clusters partitioning capture biologically meaningful structure in the dataset. To assess whether the identified clusters reflect statistically meaningful differences along the ordination axes displayed in the PCoA, we compare cluster-related first and fourth ordination scores. Both the first and the fourth axes significantly explained differences in cluster means (Kruskal–Wallis test, *p* < 0.001). Post-hoc Dunn's tests (FDR correction) showed that PC1 primarily discriminated clusters 3 from the others, while cluster 5 displayed intermediate values overlapping with clusters 1, 2 and 4. Similarly, PC4 grouped clusters 1 and 3 together and clusters 2 and 4 together, with cluster 5 showing a distinct and separate group (Figure 3e). The pairwise adonis PERMANOVA analysis further supported these distinctions: as expected all the inter-cluster comparisons were statistically significant, however, not all cluster comparisons explained the same amount of variance, as R^2^ values ranging from 0.167 to 0.311, with highest amount reported for Cluster 1 vs Cluster 2 comparison and the lowest amount reported for Cluster 1 vs Cluster 3 and 2 comparison (Figure 3c). Therefore, a hierarchical clustering analysis was performed using the Bray–Curtis dissimilarity matrix and the average linkage method. The fidelity of the resulting dendrogram to the original distance matrix (used for the unsupervised analysis) was evaluated via cophenetic correlation (r = 0.83), indicating appropriate representational accuracy. The clustering structure was reported by using a dendrogram with cluster annotations, to highlight the correspondence between ordination-based and hierarchical groupings (Supplementary Figure S5). To further highlight the distribution of OTUs based on abundance, a cluster analysis was performed using Euclidean distance and the Ward D2 linkage (Figure 3d). The analysis was conducted on the 21 OTUs whose abundance significantly (Wilcoxon test with FDR correction) changed among the 5 clusters produced by the unsupervised learning (Supplementary Figure S5). Despite the use of different cluster methods, the samples grouped coherently with the unsupervised cluster assignment, exhibiting consistent compositional profiles that confirmed the robustness of the clustering approach. Several OTUs displayed clear cluster-specific enrichment, with higher abundances concentrated in defined subsets of samples, whereas others showed a more heterogeneous distribution across clusters. Notably, low-abundance taxa substantially contributed to the observed differences, reflecting a less rich and polarized microbiota structure.

To investigate the contribution of specific OTUs to the observed community structure, we build PCoA ordination using an envfit-like approach. Statistically significant OTUs (*p* < 0.05) were then assigned to sample clusters by computing the cosine similarity between each OTU's vector of variation and the centroids of identified clusters in ordination space. This procedure relies on a geometric, vector-based association, whereby OTUs are linked to clusters based on the directional alignment and angular proximity of their variation vectors within the reduced ordination space. This vector-based method revealed strong directional associations between subsets of OTUs and specific clusters, suggesting that distinct taxonomic signatures underlie the observed microbial community patterns, independently of measured environmental or lifestyle variables (Supplementary Figure S4 and Supplementary Table S5).

To provide an overall taxonomic description of the clusters we described the most represented phyla and genera. Taxa were filtered using combined global abundance and prevalence thresholds to retain consistently represented features (Supplementary Table S6). Distinct cluster-specific taxonomic profiles emerged. Bacillota was primarily represented in clusters 3–5, Actinomycetota was mainly represented in cluster 2, while Pseudomonadota in cluster 1, with Bacteroidota showing lower and more uniform contributions (Supplementary Table S6). These patterns were mirrored by the genus-level proliferation, with *Enterococcus*, *Staphylococcus*, and *Streptococcus* characterizing clusters 3, 4, and 5, respectively; *Bifidobacterium* and *Collinsella* characterizing cluster 2; while *Escherichia* and *Sutterella* mainly represented in cluster 1, highlighting distinct bacterial community structures across clusters (Supplementary Table S6).

The differences in the relative abundances of specific OTUs among the five clusters were assessed. Considering the most representative OTUs within the dataset (see *Taxonomic signature associated with the clusters section* in the Supplementary information file), we identified 21 OTUs whose relative abundances significantly changed among the five clusters (Supplementary Figure S6). Each of these OTUs exhibited significantly different abundance patterns when compared between each cluster by using post-hoc analyses (Supplementary Table S7). Each cluster showed prevalences in specific OTUs; Cluster 1 was characterized by species-level OTUs such as *All*oprevotella *sp004555055*, *Collinsella sp900764415*, Sutterella sp90519795, *Escherichia fergusonii*, *Escherichia coli F*, and *Escherichia coli*. These OTUs were predominant within cluster 1's microbiota, although some were also shared with other clusters, for instance, *Escherichia whittamii* and *Escherichia sp0044211955*, which were also representative of cluster 2. However, cluster 2 showed a significant association in species-level OTUs belonging to the *Bifidobacterium* genus, such as *Bifidobacterium infantis*, *Bifidobacterium longum*, and *Bifidobacterium adolescentis*, the latter also being partly represented in cluster 5. Clusters 5, 4 and 3 were each associated with distinct OTU signatures that contributed substantially to their overall OTU abundance profiles. Specifically, *Streptococcus pasteurianus* was related to cluster 5, while *Staphylococcus epidermidis* was associated with cluster 4, and *Enterococcus faecalis* was associated with cluster 3. This overview of OTUs abundance variations highlights the presence of distinct microbiota types underlying each cluster, highlighting specific bacterial signatures in the meconium that appear to be independent of maternal diet and lifestyle habits.

### Assessment of maternal lifestyle factors and predicted microbial functions across cluster-defined groups and maternal-related variables

Maternal gestational age in weeks, dietary and lifestyle factors were examined to explore whether distinct clusters could be associated with specific maternal profiles. To investigate potential multivariate differences, all variables were considered for adonis PERMANOVA analysis and PCoA ordination (Figure 3f). PCoA of the joint distribution of the eight variables confirmed substantial overlap among clusters in the multidimensional space, indicating that no discrete separation emerged from the combined maternal parameters. These observations were supported by adonis PERMANOVA, which indicated no significant overall differences among clusters (R^2^ = 0.027, *p* = 0.672; Figure 3f), highlighting that the identified clusters are not related to the maternal dietary or lifestyle variables considered. Therefore, the comparison of overall patterns revealed largely overlapping profiles across clusters, with no apparent cluster-specific trends. Univariate analysis highlighted that none of the examined variables showed a statistically significant difference among the five clusters (Kruskal–Wallis, all *p* > 0.17). Despite the lack of statistical significance, the mean normalized values ​​(Figure 3g) highlight slight tendencies. Subjects exhibiting higher mean values of weight gain and end-pregnancy weight were predominantly associated with Cluster 5 (0.52 and 0.23, respectively), which also displayed the highest mean gestational age (0.68) (Figure 3g). Individuals with higher average of PA were mainly grouped in Cluster 3 (0.52), whereas participants with elevated FFQ scores, indicative of more satisfactory dietary habits, were more frequently observed in Cluster 2 (0.71) (Figure 3g). Across all clusters, the observed differences in maternal lifestyle and anthropometric variables were subtle and did not reach statistical significance (all Kruskal–Wallis *p* > 0.17), therefore the analysis highlighted only potential trends that may inform hypotheses regarding cluster-specific maternal characteristics and warrant further investigation in larger or targeted studies.

To investigate the predicted functional potential associated with the 21 bacterial OTUs whose abundance significantly differed among clusters (Supplementary Figure S6), the corresponding PICRUSt2 functional predictions were extracted and analyzed. A predictive pathway-by-OTU matrix that infers the metabolic potential of the 21 OTUs was produced, highlighting the presence of 38 “core” pathways, i.e. shared by all 21 OTUs, 227 “accessory” pathways, i.e. shared between subgroups of heterogeneous OTUs and 2 “unique” pathways present only in the OTU RS_GCF_029024925 (*E. faecalis*) among the 21 OTUs considered (Supplementary Figure S8). The predicted functional “core” metabolism was mainly composed of pathways involved in central carbon metabolism, particularly the oxidative and non-oxidative branches of the pentose phosphate pathway, together with multiple biosynthetic routes predicted for the de novo synthesis of amino acids, including aromatic, branched-chain, and polar amino acids. Pathways associated with bacterial cell wall biosynthesis, such as peptidoglycan and UDP-N-acetyl-D-glucosamine synthesis, as well as fatty acid and membrane lipid biosynthesis, were also predicted. In addition to these shared functions, the OTUs RS_GCF_029024925 (*E. faecalis*) harbored two uniquely predicted pathways, i.e. PWY-5100 and PWY-6731, relating to pyruvate fermentation to acetate and lactate II and starch degradation III respectively. Therefore, functional reconstruction of the metabolic pathways associated with the 21 cluster-defining OTUs revealed a substantial overlap across OTUs, which did not mirror the taxonomic differentiation observed in the cluster-based analyses. This finding is consistent with the limitations of the PICRUSt approach, which infers functional potential from reference genome annotations rather than directly measuring genetic content, thereby providing a lower-resolution prediction of functional variation. Consequently, while the predicted pathways offer insights into potential metabolic capabilities, they may not fully capture the subtle functional differences related to the observed taxonomic variations.

Predicted functional profiles inferred by PICRUSt2, concerning KEGG Orthologs (KOs), Enzyme Commission (EC) numbers and MetaCyc pathways, together with the observed OTU-level abundances were analyzed using MaAsLin2 to evaluate their associations with several key parameters such as gestational age, GWG, maternal age and pre-BMI. No significant associations were observed between predicted bacterial functions and GWG, maternal age or pre-BMI. However, gestational age was significantly associated with predicted bacterial functional profiles, with several significant features showing negative regression coefficients. Overall, 21 KOs, 9 EC numbers and one pathway (P221-PWY, octane oxidation pathway) remained significant after false discovery rate correction (*q* < 0.05) (Figure S7 and Table S8). The associated KOs and EC numbers were predominantly related to sulfur metabolism, oxidoreductase activity, amino acid metabolism and bacterial stress-response functions. However, these predicted functional features were present in only 5.3%–16.7% of the analyzed samples, indicating that they represent relatively low-prevalence functional signatures rather than widespread functional characteristics of the study population.

The MaAsLin2 analysis, using the same model described for the predictive functional profiles analysis, on the observed bacterial OTUs identified a single significant association after multiple-testing correction. The abundance of OTU RS_GCF_002269255.1 (*Klebsiella quasivariicola*) was negatively associated with gestational age (*q *= 0.043), indicating a progressive decrease in its observed relative abundance with increasing gestational age. Whereas no significant associations were detected for GWG, maternal age or pre-BMI. Similar to the predicted functional findings, this OTU was present in only 7 of the 132 study participants, i.e. 5.3%, suggesting that the observed taxonomic association was restricted to a limited subset of the study cohort.

## Discussion

In the current study, we investigated the influence of maternal GWG and other maternal lifestyle and physiological variables on the composition of neonatal meconium microbiota within the LIMIT cohort,^13^ applying a long-read sequencing approach to provide high-resolution microbial profiling. According to the inclusion and exclusion criteria of the LIMIT protocol,^13^ the study population was restricted to physiological pregnancies and newborns not requiring intensive care.

Our analyses revealed that neither GWG categories nor maternal pre-BMI, MD adherence, or PA, exerted a measurable effect on meconium microbial diversity. These findings suggest that maternal weight gain and lifestyle-related factors, although crucial for pregnancy outcomes and child health, may not directly shape the earliest microbial communities in newborns. The results are in line with previous work of Turunen et al.^3^ which pointed to the delivery mode as the strongest determinant of meconium microbial composition, thereby reinforcing the idea that gut microbiota establishment begins primarily at birth through exposures occurring during delivery and in the immediate postnatal period. Therefore, the lack of significant microbial community variation in meconium samples associated with GWG categories and other maternal factors, such as pre-BMI, MD adherence, and PA level, suggests that the initial establishment of the neonatal gut microbiota may be driven by complex, multifactorial processes not directly explained by the maternal lifestyle variables measured in this study.

At the same time, our results stand in contrast to other studies^36^ ^,^ ^37^ reporting significant associations between maternal GWG and meconium microbiota. For instance, evidence from Song and Liu^36^ found that EGWG was linked to reduced α-diversity and depletion of specific microbial taxa. Another study conducted by Cho et al.^37^ exploring the relationship between maternal pre-BMI, GWG, and early gut colonization, revealed that maternal pre-BMI was associated with distinct microbial profiles in neonatal meconium. *Citrobacter* was more abundant in infants of mothers with normal pre-BMI, while higher *Lachnospira* levels were found in newborns of mothers experiencing underweight before pregnancy.

Additionally, maternal GWG was found to be linked to specific microbial signatures in neonatal meconium, with variations in bacterial composition observed across different levels of maternal weight gain.^37^ Discrepancies across studies may reflect differences in cohort characteristics and study design, including population background, maternal metabolic profile and dietary habits. In addition, variation in eligibility criteria, sampling time points, microbiome profiling pipelines, and statistical modelling may influence the associations reported. Our study also considered maternal dietary patterns and lifestyle variables. Although maternal adherence to the MD and reported dietary habits showed no significant associations with meconium microbial diversity, this does not rule out their importance. Prior work by Sasaki et al.^7^ demonstrated that higher adherence to the MD during pregnancy correlated with increased abundance of SCFAs-producing bacteria in meconium, with epigenetic changes in placental and cord blood tissues. The lack of associations in our cohort may be explained by methodological differences, by the exclusion of alcohol from the MD score during pregnancy, or by the fact that dietary influences may become more evident later in infancy during breastfeeding and complementary feeding, when maternal diet exerts a more direct impact. Similarly, no significant relationships were observed for maternal PA or smoking status, though these behaviors remain important for overall pregnancy and infant health. One of the strengths of this study lies in its use of unsupervised clustering, using the PAM algorithm, to move beyond predefined maternal categories and explore intrinsic microbial structures. This approach revealed that neonatal meconium harbors distinct microbial community types. The identification of five clusters, each dominated by characteristic taxa such as *Escherichia* and *Sutterella* in one cluster, *Bifidobacterium* and *Collinsella* in another, and Bacillota-associated genera like *Enterococcus*, *Staphylococcus*, and *Streptococcus* in others, suggests that early-life gut colonization may already follow divergent trajectories. These patterns appear largely independent of maternal dietary or lifestyle factors, emphasizing that meconium represents a unique and largely intrinsic microbial substrate. The presence of cluster-specific OTUs further supports the concept of early differentiation of the neonatal microbiota, which may set the stage for subsequent microbiota development and functional specialization. These findings suggested that meconium “types” or enterotype-like structures, reminiscent of clustering observed in adult gut microbiota studies, align with the results of Chang et al.^38^ who reported similar microbial community structures in meconium using long-read sequencing.

Overall, although derived from a predictive approach, the PICRUSt2-based analysis suggests that the meconium-associated microbiota may possess metabolic potential characterized by substantial biosynthetic autonomy. The predominance of predicted pathways involved in de novo amino acid biosynthesis and central carbon metabolism is consistent with an adaptation to a nutrient-limited environment such as meconium, which represents a transient niche during the early stages of neonatal intestinal colonization. In this context, the presence of uniquely predicted pathways in the RS_GCF_029024925 genome may indicate functional specialization, potentially related to maternal dynamics or early postnatal exposure that we cannot currently explain. However, these interpretations require a high degree of caution, as they rely solely on functional inference from 16S rRNA gene sequencing data, which does not provide direct evidence of the gene's presence, expression, or metabolic activity.

It is important to note that these clusters were largely independent of maternal dietary, anthropometric, and lifestyle factors, as indicated by both multivariate and univariate analyses. However, some inferences between maternal parameters and clusters are interesting, although speculative due to their trends and lack of significance. The identification of latent bacterial structures through unsupervised learning highlights early differentiation of the neonatal microbiota, which could have implications for subsequent microbiota development and potential functional specialization. These inferences include, for example, the increased maternal weight gain with clusters enriched in Bacillota (also called Firmicutes) or a higher food frequency score with clusters characterized by *Bifidobacterium*. However, the lack of significant relationship, corroborates the intrinsic nature of the neonatal meconium microbiota composition regardless of the maternal factors considered in the study.

From an inferential functional perspective, the Bacillota-dominated clusters (clusters 3–5) include genera known for producing lactic acid (*Streptococcus*, *Enterococcus*), which may modulate intestinal pH and barrier function^39^ while cluster 2, enriched by *Bifidobacterium*, may support early carbohydrate metabolism and the production of SCFAs or other beneficial bioactive compounds.^40^ ^,^ ^41^ *Bifidobacterium* species, particularly *B. longum subsp. infantis*, were also known to efficiently metabolize human milk oligosaccharides and to promote the production of bioactive metabolites involved in immune regulation and gut barrier maturation.^42^ Previous evidence indicates that *Bifidobacterium*-dominated early-life microbiota can support immune priming by reducing pro-inflammatory responses and promoting regulatory pathways, potentially reducing the risk of immune-mediated disorders later in life.^42^ Although functional data were not directly assessed in this study, the predominance of *Bifidobacterium* species (*B. longum*, *B. infantis* and *B. adolescentis*) in cluster 2 suggested a bacterial profile that may favor less disorder-prone immune and metabolic development during the neonatal period.

The supervised multivariable modelling performed using MaAsLin2 highlighted that gestational age emerged as the only maternal variable consistently associated with the predicted functional profile of the gut microbiota. Although the identified KOs and EC numbers encompassed diverse biological functions, many converged-on oxidoreductase activities and sulfur metabolism, suggesting changes in the predicted redox-related metabolic potential rather than isolated functional alterations. Sulfur-transforming pathways and oxidoreductase enzymes are fundamental components of bacterial physiology, contributing to electron transfer, maintenance of intracellular redox balance and adaptation to anaerobic environments.^43^ Notably, all significant associations showed negative coefficients, indicating a progressive decline in the predicted abundance of these functions with advancing gestational age, suggesting a gradual remodeling of the predicted functional landscape of the neonatal gut microbiota throughout pregnancy.

Interestingly, whereas only a single bacterial OTU was significantly associated with gestational age, multiple predicted functional features remained significant after multivariable adjustment. This apparent discrepancy can be related to a functional redundancy mechanism, whereby phylogenetically distinct bacteria encode overlapping metabolic capabilities, allowing bacterial communities to preserve similar functional potential despite changes in taxonomic composition. At the same time, the coordinated decline of a subset of predicted redox-related functions suggests that specific components of the neonatal gut bacterial functional repertoire may progressively change during gestation.

Therefore, it should be noted that the significant predicted functional features were detected in only a relatively small proportion of the study population, indicating that these associations likely characterize specific subsets of individuals rather than representing overall functional traits of the neonatal gut microbiota. Moreover, as previously mentioned, the predicted functional profiles analyzed in this study were inferred using PICRUSt2 and therefore represent predicted functional potential based on reference genome annotations rather than directly measured gene content or metabolic activity. Consequently, while these findings provide valuable insights into the potential functional contribution of the neonatal gut microbiota in relation to gestational age, confirmation through more direct approaches will be necessary to validate these predicted functional changes and to determine whether they translate into measurable differences in bacterial metabolic activity.

Overall, these findings suggest that there are underlying ecological states of the neonatal gut microbiota, the functional potential of which requires further exploration, including how they evolve in the postnatal period and whether they predict neonatal health outcomes, including growth patterns, immune development, and susceptibility to metabolic or allergic conditions. As mentioned previously, these aspects, which were not sufficiently defined by the functional prediction analysis (PICRUSt2), are among the limitations of the study and require future investigation.

Therefore, our findings supported the notion that early-life bacterial colonization may follow distinct ecological trajectories, potentially shaped by perinatal exposures, genetic predispositions, or stochastic events during the gestational period and delivery, rather than specific maternal lifestyle or nutritional status. The emergence of defined microbiota configurations at birth conceptually aligns with enterotype-like structures described in adult gut microbiota,^44^ ^,^ ^45^ where discrete community states have been proposed. At the same time, the origin and biological significance of these early bacterial patterns remain incompletely understood. For instance, several studies have questioned the existence of a prenatal microbiome.^46^ ^,^ ^47^ For example, Kennedy et al.^46^ reported no evidence of microbial presence in 20 prenatal meconium samples collected under sterile conditions, supporting the hypothesis that initial gut colonization may initiate only during or after delivery.^46^ Similarly, Santos Scott et al.^47^ reported difficulties in identifying a distinct microbiome in 141 meconium samples from which DNA was extracted, also under sterile conditions to avoid contamination.^47^ These divergent findings may be due to differences in DNA extraction and sequencing protocols, contamination controls, or sensitivity of detection, and they underline the need for methodological rigor and standardization in future research.

In this context, our findings do not aim to resolve the debate on prenatal colonization, but rather to describe the existence of bacterial patterns detected at birth and explore their potential ecological and functional relevance. Specifically, the consistent identification of discrete and cluster-specific taxonomic signatures in samples suggests that the neonatal gut microbiota may already be structured into distinct bacterial configurations before birth.

The application of unsupervised learning approaches in this context proved crucial for uncovering meaningful variation that would otherwise have remained hidden. This has significant implications for the study of microbiota development, highlighting the need for refined methodologies capable of capturing the complexity of host-microbe development in early life.

Limitations of this study must also be acknowledged. The analysis was restricted to meconium collected at birth, representing a single time point and therefore unable to capture the dynamic colonization processes that occur immediately after delivery and during early infancy. Consequently, the present cross-sectional design does not allow us to determine whether the bacterial clusters identified in meconium persist over time, predict subsequent microbiome trajectories, or influence later health outcomes. Therefore, our findings should be interpreted as descriptive of initial microbial patterns at birth rather than as indicators of long-term microbiome development. The sample size, while larger than many previous studies, may still have been insufficient to detect subtle associations between maternal variables and bacterial structure. In addition, the use of composite dietary indicators such as the MEDI-LITE score,^19^ although conceptually justified, may have limited the resolution of dietary exposure assessment. Although caesarean-delivered samples were excluded to minimize major confounding, other unmeasured factors, including maternal stress, environmental microbial exposures, or technical variability, may have contributed to inter-individual microbial differences.

Despite these limitations, our findings provide important insights into the complex and multifactorial nature of meconium microbiota establishment. The identification of distinct, biologically coherent microbial clusters in meconium suggests that neonatal gut colonization may follow predefined ecological trajectories already detectable at birth. Future research should therefore adopt a longitudinal perspective, integrating repeated sampling and multi-omics framework, across the first 1000 d of life. The longitudinal design of the LIMIT cohort^13^ will provide a unique opportunity to determine whether these meconium-associated microbiota types persist, converge, or diverge over time, and whether they represent a predictive of later outcomes, including growth trajectories, immune maturation, and susceptibility to metabolic or inflammatory conditions.

In conclusion, while maternal gestational and lifestyle variables did not significantly explain meconium microbiota variability, unsupervised learning revealed discrete and biologically relevant microbiota types in newborns' meconium. These findings suggest that neonatal microbial colonization may be influenced by stochastic processes, including founder effects and ecological drift, as well as subtle perinatal exposures (e.g., differences in timing or order of initial contacts with maternal skin and vaginal or rectal microbiota or delivery-room microbes), which may outweigh broader maternal influences at this early stage.^48-50^ These results underscore the intrinsic and complex nature of early microbial colonization and highlight the value of data-driven approaches in uncovering latent biological structures that may be critical for understanding early-life microbiota development. The complete (1000 d) longitudinal dataset will be crucial to disentangle the determinants shaping these early bacterial profiles and to determine whether such initial community configurations persist, evolve, or influence subsequent microbiota development and early-life health trajectories.

## Supplementary Material

Supplementary material Supplementary Table 7.csv

Supplementary material Supplementary Figure 1.tiff

Supplementary material Supplementary Figure 2.tiff

Supplementary material Supplementary Figure 3.tiff

Supplementary material Supplementary Figure 4.tiff

Supplementary material Supplementary Figure 5.tiff

Supplementary material Supplementary Figure 6.tiff

Supplementary material Supplementary Figure 7.tiff

Supplementary material Supplementary Figure 8.tiff

El_Masri_Supplementary_file_II_final.docx El_Masri_Supplementary_file_II_final.docx

## Data Availability

Raw-derived data and metadata files required to reproduce the statistical analyses reported in this study, along with the detailed overview table showing the output of library preparation and sample sequencing, are openly available in the updated version (January 27, 2026) of the study database present in the Zenodo repository at the following DOI: https://doi.org/10.5281/zenodo.18376726. All results from statistical analyses were mentioned by writing in the main text, represented as figures in the main text or reported as Supplementary Tables and Figures. Further information can be provided upon reasonable request to the corresponding author.
