# Supplementary material for: Early-life microbiota in the LIMIT cohort: unveiling meconium microbiota types beyond maternal lifestyle
Source: Gut Microbes. 2026 Aug 4;18(1):2712811. doi: 10.1080/19490976.2026.2712811 (PMC13449723; doi:10.1080/19490976.2026.2712811)
Supplement: Supplementary file 1 [file KGMI_A_2712811_SM1560.docx]

**Supplementary information**

**Early-life microbiota in the LIMIT cohort: unveiling meconium microbiota types beyond maternal lifestyle**

**Dana El Masri^1^*, Niccolò Meriggi^2^*, Federica Loperfido^1^, Irene Bianco^1^, Chiara Ferrara^1^, Beatrice Maccarini^1^, Francesca Sottotetti^1^, Carlotta De Filippo^2^, Nicolò Loddo^3^, Rosa Maria Cerbo^3^, Stefano Ghirardello^3^, Francesca Garofoli^3^, Micol Angelini^3^, Maria Cristina Monti^4^, Mulubirhan Assefa Alemayohu^4^, Mie Bech Lukassen^6^, Hellas Cena^1,5§^, Rachele De Giuseppe^1§^.**

*El Masri D and Meriggi N, same contribution

^§^Cena H and De Giuseppe R, same contribution

^1^ Laboratory of Dietetics and Clinical Nutrition, Department of Public Health, Experimental and Forensic Medicine, University of Pavia, 27100 Pavia, Italy

^2^ Institute of Agricultural Biology and Biotechnology (IBBA), National Research Council (CNR), 56124 Pisa, Italy

^3^ Neonatal Unit and Neonatal Intensive Care Unit, Fondazione IRCCS Policlinico San Matteo, 27100 Pavia, Italy

^4^ Biostatistics and Clinical Epidemiology Unit, Department of Public Health, Experimental and Forensic Medicine, University of Pavia, 27100 Pavia, Italy

^5^ Clinical Nutrition and Dietetics Service, Unit of Internal Medicine and Endocrinology, ICS Maugeri IRCCS, 27100 Pavia, Italy

**Corresponding Author**

**Federica Loperfido, MSc**

Laboratory of Dietetics and Clinical Nutrition, Department of Public Health, Experimental and Forensic Medicine, University of Pavia, 27100 Pavia, Italy

e-mail: [federica.loperfido@unipv.it](mailto:federica.loperfido@unipv.it)

*Dataset quality assessment*

The DNA concentration was quantified using the Qubit fluorometric assay, ranging from 0 (not DNA extracted) to 81.1 ng/µL across 183 samples (median = 1.61 ng/µL; mean = 5.02 ng/µL), consistent with the low-biomass nature of meconium samples. DNA extraction and sequencing library preparation was successful for 168 / 183 sample analyses (91.8 %), and yielded between 2’688 and 126’205 DNA reads after QC and bioinformatic processing. Failed samples were those yielding significantly fewer mapped DNA reads (mappedReads) than 10’000; here mappedReads < 2’000. Low-read samples were disregarded in all subsequent analyses. During metagenomic library preparation, from dsDNA extraction through library sequencing, dsDNA concentrations were quantified for each sample and the related sequencing outputs were systematically monitored. Specifically, the numbers of raw, quality-filtered, and mapped reads generated for each sample were recorded and summarized in a comprehensive overview table (*summary_table*, available in the Zenodo repository at the following DOI: https://doi.org/10.5281/zenodo.18376726).

Read retention was evaluated across the main processing steps from raw reads to final mapped reads. On average, approximately 4.3% of reads were lost during quality filtering, i.e. reads passing quality filtering and reads successfully mapped to reference compared to the raw reads, while the loss relative to raw reads reached around 10.4% at the final mapping step.

The bioinformatic workflow produced an OTU reference sequences file in FASTA format, loaded using the “Biostrings” package v2.70.3.^1^ Several sequences were flagged for containing non-standard nucleotide characters outside the IUPAC DNA alphabet. These sequences were inspected, then, to preserve biological interpretability while ensuring compatibility with downstream functions, non-IUPAC characters were removed, resulting in a cleaned FASTA format sequence object, combined into an ampvis2 object using the “ampvis2” package v2.8.9,^2^ together with the OTU count data table and metadata from the study cohort.

After quality filtering, the dataset was composed of 168 samples, of 12’706’791 reads (minimum: 2’688, maximum: 126’205, median: 87’214) collapsed in a total of 538 OTUs. To explore community structure and sequencing depth adequacy, both rank-abundance and rarefaction curves were generated using the R package “ampvis2”, highlighting that sequencing was effective in adequately sampling the microbial diversity (Supplementary Figure S1). Moreover, the total number of reads did not differ across the main study variables, excluding sequencing-related biases and validating the subsequent statistical analyses (Supplementary Figure S2). To provide an overall description of the entire dataset, we described the main bacterial classes and species, reporting the percentage of total sequencing reads across the entire dataset. The meconium microbiota appears to be less heterogeneous than expected, characterized by a low number of bacterial classes that together represent almost the entire total percentage of sequencing reads. The five most represented classes accounted for 95.3% of total sequencing reads. Bacilli represent the most represented class (5’435’046 reads; 42.8%), followed by Gammaproteobacteria (3’295’887 reads; 25.9%), Actinomycetes (1,522,212 reads; 12.0%), Coriobacteriia (1’042’026 reads; 8.2%), and Bacteroidia (812’372 reads; 6.4%). At the species level, the five most represented species together accounted for approximately half of total sequencing reads (49.4%), indicating a community structure characterized by a limited number of dominant bacterial species. The most represented species was *Enterococcus faecalis* (2’789’896 reads; 22.0%), followed by *Sutterella sp. 905197905* (1’352’769 reads; 10.6%) and *Staphylococcus epidermidis* (935’647 reads; 7.4%). *Collinsella sp. 902469435* (622’326 reads; 4.9%) and *Streptococcus pasteurianus* (570’209 reads; 4.5%).

**Supplementary Figure 1**. (a) Rank-abundance curves showing the abundance percentage of OTUs ranked by decreasing abundance across samples grouped by GWG categories. The x-axis represents the rank of each OTU (following abundance rank), while the y-axis shows the corresponding abundance percentage on a linear scale. Shaded areas indicate standard deviations across replicates within each group. (b) Rarefaction curves depicting the relationship between sequencing depth (total number of reads) on the x-axis and observed OTU richness (total number of OTUs) on the y-axis, for each sample, coloured according to GWG categories. The step size for calculating rarefaction points was set to 50 reads, and the x-axis was constrained to a maximum of 5’000 reads.

**Supplementary Figure 2**. Boxplot reports the sequencing depth expressed as logarithm of the total number of reads per sample among the main categorical variables considered in the study. Differences are assessed using an ANOVA test after fitting the model formula using a linear model. P-values from the analysis are reported on top of each panel. All the categories within each variable in which the metadata are not annotated during enrollment are indicated as not reported (nr).

To properly perform the downstream statistical analysis according to the study project's purposes, the dataset underwent a cleaning process illustrated as follows. We kept only the subjects that presented a category within the GWG variable, as the main variable considered in the study, thus eliminating 13 samples with no GWG reference, keeping a total of 155 samples with 531 associated OTUs. The birth type was initially included in the permutation model, showing a significant effect (Adonis PERMANOVA: R^2^ = 0.02, p < 0.001). This was likely attributable to both the imbalance in the dataset as the c-section samples were 23 against 132 samples with normal birth and for a real biological effect of delivery mode on meconium microbiota as already documented.^3^ To reduce the bias resulting from the dataset imbalance, a permutation analysis was performed by increasing the number of permutations to 9’999 and stratifying within the birth type, that is, the birth type was set as a group within which to constrain permutations. This analysis highlights a significant effect albeit reduced (Adonis PERMANOVA: R^2^ = 0.02, p = 0.04). In line with this evidence and the aim of the study, all subsequent analyses were conducted exclusively on samples from individuals born via natural delivery and with an assigned classification within the GWG category. Therefore, samples related to c-section delivery mode and lacking GWG information were excluded to minimise potential confounding effects that could lead to misleading interpretations. After this further quality filtering, the dataset was composed of 132 samples, of 10’246’006 rds (minimum: 2’688, maximum: 126’205, median: 96’442.5) collapsed in a total of 436 OTUs. To provide an overall description of the entire dataset after c-section samples removal, we described again the main bacterial classes and genus, reporting the percentage of total sequencing reads across the entire dataset. At the class level, taxonomic composition continues to be dominated by a small number of major classes, in fact the five most represented classes accounted for approximately 95.3% reads calculated as the proportion of total sequencing reads across the dataset. Bacilli was the most represented class (4'198'020 reads; 41.0%), followed by Gammaproteobacteria (2'573'201 reads; 25.1%), Actinomycetes (1'262'875 reads; 12.3%), Coriobacteriia (1'003'257 reads; 9.8%), and Bacteroidia (726'414 reads; 7.1%). At the species level, the ten most abundant species together accounted for approximately 50.5% of total sequencing reads. The most represented species was *Enterococcus faecalis* (2'358'930 reads; 23.0%), followed by *Sutterella sp. 905197905* (1'144'023 reads; 11.2%), *Staphylococcus epidermidis* (639'436 reads; 6.2%), *Collinsella sp. 905197905* (600'527 reads; 5.9%) and *Alloprevotella sp. 004555055* (487'325 reads; 4.8%).

The study of c-section subjects was beyond the scope of this study, and given the samples imbalance which makes the analyses prone to statistical bias, the significant effects of c-section delivery on meconium bacterial diversity as previously mentioned, we removed these samples from the dataset to avoid confounding effects. However, we further investigated this comparison to provide analytical support for the differences observed between main taxonomic groups of c-section and natural birth delivery type. Relative abundances percentage of bacterial families were calculated for each sample by summing the read counts of all OTUs assigned to the same family and normalizing by the total reads per sample, expressed as a percentage. Families with missing or unassigned taxonomy were excluded. To test for differences in relative abundance between birth types (c-section vs. natural delivery), we performed the Kruskal-Wallis test for each family individually. Only families with a p-value < 0.05 were considered significant and included in supplementary figure 3.

Analysis identified variations in relative abundance percentages in several families, in total 22 bacterial families showed relative abundance significantly differed between infants delivered via c-section or natural (Kruskal-Wallis test, p < 0.05; Figure S3). Specifically, Bacteroidaceae, Bifidobacteriaceae, Burkholderiaceae, Coriobacteriaceae, Enterobacteriaceae, Microbacteriaceae, and Anaplasmataceae were significantly more abundant in naturally delivered infants, whereas all other families, including CAG-314, CAG-508, Coprobacteraceae, Dialisteraceae, Marinifilaceae, Oscillospiraceae, Rikenellaceae, Ruminococcaceae, UBA1820, UBA660, Akkermansiaceae, Acetonemaceae, Acutalibacteraceae, Anaerovoracaceae, Barnesiellaceae, were significantly more abundant in c-section delivered infants (Supplementary Figure S3). These differences confirm that birth mode influences early colonization patterns in the meconium microbiota, however we reiterate that this data is subject to dataset imbalance conditions.

**Supplementary figure 3.** Relative abundance of bacterial families significantly associated with birth mode. Bar plots show the mean relative abundance (%) ± standard error for families that were significantly different between c-section and natural delivery infants (Kruskal–Wallis test, p < 0.05). Birth type is reported using color code in the legend.

A further sample with missing values in key metadata variables (i.e. GWG, child gender, breastfeeding, pre-pregnancy BMI, pre-pregnancy weight, weight gain, MEDI-LITE score, medi lite score rank, PA, DH, FFQ and IPAQ) was also excluded to ensure complete case analysis and improve the robustness and interpretability of downstream multivariate statistical analyses, obtaining a final clean dataset of 131 samples suitable for all subsequent analyses. The adonis PERMANOVA analyses described were carried out by using the *adonis2* function of “vegan” package v2.6.10,^4^ based on Bray-Curtis distance matrix made from normalised counts (relative abundance).

*Bacterial diversity analysis according to the mother’s diet and lifestyle variables*

Performing the *Adonis2* function by using a model formula which include several factors and potentially collinear variables could affect the outcome of the analysis, so an analysis to test the potential collinearity was first performed by using Variance Inflation Factor (VIF) analysis, to remove predictors with collinearity and select an appropriate number of factors to include in the model formula of permutational multivariate analysis. This analysis was performed by using the *vif* function of the "car" package.^5^ We considered as potentially collinear variables those with a Generalised VIF (GVIF) value > 5; among these, we identified the pre-pregnancy BMI and pre-pregnancy weight variables, which reported a GVIF value of 6.37 and 5.49, respectively, and their correlation was also tested (Pearson; corr: 0.894, p < 0.001). Therefore, we exclude the pre-pregnancy weight from the model formula. Therefore, the Adonis PERMANOVA analysis was carried out, producing the output reported in Supplementary Table S1, highlighting the effect of the main variables selected on the bacterial diversity. The Adonis PERMANOVA analysis was performed on the Bray-Curtis distance matrix produced from normalised count (relative abundance) and setting 1’000 number of permutations.

**Supplementary Table 1**. Adonis PERMANOVA table. The table reports the results of the analysis of Adonis PERMANOVA. Factors considered in the model formula are reported with the related Adonis output (Sum of Squares, R^2^ and P-value). Continuous variables (pre-pregnancy BMI, PA, DH, FFQ, IPAQ) are added in the model as covariates.

| Factor | SumOfSqs | R2 | P-value |
| --- | --- | --- | --- |
| GWG | 0.541 | 0.011 | 0.8591 |
| Child gender | 0.235 | 0.005 | 0.8591 |
| Breastfeeding | 1.211 | 0.024 | 0.3696 |
| Pre pregnancy BMI | 0.371 | 0.008 | 0.4835 |
| Weight gain | 0.2847 | 0.006 | 0.7273 |
| Medi lite score | 0.359 | 0.007 | 0.5345 |
| Medi lite score rank | 0.226 | 0.005 | 0.9051 |
| Mother celiac condition | 0.953 | 0.019 | 0.1728 |
| PA | 0.376 | 0.008 | 0.4555 |
| DH | 0.304 | 0.006 | 0.7013 |
| FFQ | 0.230 | 0.005 | 0.8711 |
| IPAQ | 0.351 | 0.007 | 0.5135 |
| Residual | 44.181 | 0.89 |  |
| Total | 49.626 | 1 |  |

To deeply inspect the relationship between bacterial diversity and continuous variables of nutritional and clinical data, the environmental fitting analysis was performed by using the *envfit* function of the "vegan" package v2.6.10. The analysis was performed on relative abundances -transformed counts and converted into a distance matrix using the Bray-Curtis index, then the principal coordinate axes were calculated using the *cmdscale* function. The continuous variables related to diet and lifestyle were normalized by using z-score transformation to remove scale bias which can affect the distance-based analysis. The analysis was carried out setting 1’000 numbers of permutations. Environmental fitting analysis does not reveal significant correlations between diversity and the tested variables, confirming the absence of significant effects of these variables on the bacterial diversity (Supplementary Table S2). We tested the mode of delivery (spontaneous or induced) by permutation analysis and there were no significant effects on meconium bacterial diversity (Adonis PERMANOVA: R^2^ = 0.01, p > 0.05). We also considered side parameters such as maternal age, gestational age (in weeks), and infant length and width, by performing the adonis PERMANOVA analysis, and no significant effects on meconium diversity was observed, with overall R^2^ values ranging from 0.004 to 0.007 and all p-values > 0.59. The distribution of the samples according to the biometric data of the children (weight and length) was carried out by Principal Coordinates Analysis (PCoA), represented in figure 2d.

**Supplementary Table 2**. The table reports the results from environmental fitting analysis considering the main variables related to diet and lifestyle conditions. Each vector is reported together with the related R² and p-values from permutation testing.

| Factor | R2 | Pr(>r) |
| --- | --- | --- |
| Pre-pregnancy weight | 0.017 | 0.3476 |
| End pregnancy weight | 0.010 | 0.5065 |
| Weight gain | 0.008 | 0.5994 |
| Pre-pregnancy BMI | 0.025 | 0.1838 |
| End pregnancy BMI | 0.018 | 0.3127 |
| FFQ | 0.001 | 0.9451 |
| DH | 0.002 | 0.9031 |
| PA | 0.024 | 0.2268 |
| Medi lite score | 0.011 | 0.4815 |

To assess differences in bacterial diversity (β-diversity) between GWG groups, a pairwise Adonis PERMANOVA was carried out. For each pairwise comparison, the subset of the distance matrix corresponding to the two groups (see Group 1 and 2 in Supplementary Table S3) was extracted, and a PERMANOVA test was performed using the *adonis2* function from the “vegan” package v2.6.10, setting 1’000 number of permutations. The analysis was performed on the Bray-Curtis distance matrix produced from a normalised (relative abundance) count table. To facilitate the interpretation, the resulting R² values, indicating the proportion of variance explained by group membership, were displayed as a heatmap in Figure 1b.

**Supplementary Table 3**. Table reports the results from pairwise adonis PERMANOVA testing differences between different GWG categories. For each comparison, the proportion of variance explained (R²) and the associated p-value are reported.

| **Group 1** | **Group 2** | **R^2^** | **P-value** |
| --- | --- | --- | --- |
| LGWG | AGWG | 0.006 | 0.849 |
| LGWG | EGWG | 0.008 | 0.903 |
| AGWG | EGWG | 0.013 | 0.466 |

The α-diversity metrics, i.e. Shannon, Simpson, and inverse Simpson indices, were generated for each sample using the *amp_alphadiv* function of the "ampvis2" package. Differences in α-diversity among GWG categories were assessed using Kruskal-Wallis test for each index. Differences in α-diversity among GWG categories were assessed using the Kruskal-Wallis test for each index. No statistically significant differences in α-diversity were observed between GWG groups (Shannon: p = 0.349, Simpson: p = 0.594, and inverse Simpson: p = 0.594).

*An unsupervised learning approach highlights the hidden structure of meconium microbiota*

To identify latent structure in the multivariate space, unsupervised learning was carried out by using the Partitioning Around Medoids (PAM) clustering algorithm implemented in the *pam* function of the “cluster” package v2.1.4,^6^ using the Bray-Curtis dissimilarity matrix on relative abundance transformed counts as input. The optimal number of clusters (k) was empirically determined by evaluating the average silhouette width (ASW) for k ranging from 2 to 10. ASW values were computed iteratively, and the value of k maximising mean silhouette width was selected as the optimal clustering solution. Clustering performance using the PAM algorithm was evaluated based on the minimisation of the objective function, which reflects the total dissimilarity between observations and their assigned medoids. The initial configuration, generated by the "build" phase, yielded a dissimilarity value of 0.53, and subsequent optimisation through the "swap" phase reduced this value to 0.52. The internal cohesion and external separation for each cluster were reported in Supplementary Table S4. Cluster sizes ranged from 12 (Cluster 5) to 49 (Cluster 1). The average within-cluster dissimilarities varied between 0.54 (Cluster 3) and 0.79 (Cluster 4), with lower values suggesting higher internal similarity. Notably, Cluster 3 exhibited the lowest average dissimilarity, indicating tight grouping of its members, whereas Cluster 4 reported the highest average dissimilarity, suggesting greater internal variability. To identify the most and least common clusters, the percentage of samples belonging to each cluster within the total of samples in the dataset, was calculated by dividing the number of samples assigned to that cluster by the total number of samples included in the analysis, then expressed as percentage. All the percentages associated with each cluster were reported in the Supplementary Table S4 (Frequence).

**Supplementary Table 4**. The table reports, for each cluster, the number of observations (Cluster size), maximum within-cluster dissimilarity (Max diss), average within-cluster dissimilarity (Av diss), and minimum separation from other clusters (Separation), percentage of samples assigned to each cluster relative to the total number of samples included in the analysis (Frequence).

| Cluster | Cluster size | Max diss | Av diss | Separation | Frequence |
| --- | --- | --- | --- | --- | --- |
| 1 | 49 | 1 | 0.55 | 0.22 | 37.4 |
| 2 | 19 | 0.83 | 0.58 | 0.22 | 14.5 |
| 3 | 34 | 0.99 | 0.38 | 0.24 | 26.0 |
| 4 | 17 | 0.99 | 0.62 | 0.24 | 13.0 |
| 5 | 12 | 0.99 | 0.50 | 0.38 | 9.2 |

Cluster membership was then overlaid on the first two axes of PCoA space for visualisation and interpretation. Additionally, PCoA results were extended to four dimensions to capture a higher proportion of total β-diversity variance and to evaluate inter-cluster separation along supplement ordination axes (i.e. PCo1 vs PCo4 in figure 2b). The effect of Cluster in explaining bacterial diversity was statistically assessed by using permutational multivariate analysis adonis PERMANOVA using the *adonis2* function of “vegan” package v2.6.10, setting 1’000 as the number of permutations. The adonis PERMANOVA highlights that the identified clusters explained a significant proportion of the variance in microbial community composition that reflects biologically relevant meaning in the dataset (R² = 0.35, p = 0.001). To highlight differences among clusters in multivariate ordination space, we also performed cluster-wise comparison of coordinates 1 and 4 (Figure 3e). Univariate differences among clusters were tested using the Kruskal-Wallis test, then pairwise comparisons between clusters were performed using Dunn’s post-hoc test with FDR adjustment. We observed a significant effect of cluster membership on PC1 (Chi-sqr = 77.79, df = 4, p < 0.001) and PC4 (Chi-sqr = 49.67, df = 4, p < 0.001), as both represented the first and last axis (thus the highest and lower amount of variance) used to depict the diversity in the multidimensional space, confirming that both axis was effective in discriminating the main differences among clusters. Post-hoc Dunn’s test (FDR correction) indicated that indicate that mean PC1 scores differ mainly between clusters 3 and 4 and the remaining clusters, with cluster 5 showing intermediate values overlapping clusters 1 and 3, whereas mean PC4 scores separate clusters 1 and 3 as one group, clusters 2 and 4 as a second group, and identify cluster 5 as a distinct group (Figure 3e).

To define the influence of the main OTUs on the separation of the clusters, we applied a constrained environmental fitting approach on the ordination space. The OTU relative abundance matrix was sequentially filtered to first remove OTUs with zero variance across all samples and then OTUs present in less than 5% of the samples, reducing noise from rare taxa. A PCoA was then performed based on a Bray-curtis distance matrix using the filtered community composition data described above. Significant OTUs were identified by fitting OTU vectors onto the PCoA ordination using the *envfit* function from the "vegan" package v2.6.10, setting 1’000 permutations to assess statistical significance. Only OTUs with a permutation-based p-value < 0.05 were retained and projected as vectors in the multidimensional space (Figure S4a).

The orientation of each OTU reflects its maximal correlation with the ordination axes. To associate OTUs with specific bacterial clusters, we calculated the cosine similarity between each significant OTU vector and the centroids of predefined sample clusters, defined as the mean coordinates on the first two ordination axes. Each OTU was assigned to the cluster whose centroid exhibited the highest directional similarity, providing an interpretable link between environmental fitting analysis and cluster-associated OTUs (Supplementary Figure S4b and Supplementary Table S5). The cosine similarity formula was: cos(θ) = A⋅B / ∥A∥ ∥B∥; where A⋅B is the dot product of the two vectors, and ∥A∥ and ∥B∥ are their euclidean norms. This metric quantifies the cosine of the angle (θ) between the vectors, with values closer to 1 indicating higher similarity in direction.

**Supplementary Figure 4**. OTUs-Clusters association using cosine similarity. (a) PCoA (Bray-Curtis distance) reports the sample distribution according to the clusters produced by the PAM algorithm. The OTUs significantly selected by environmental fitting analysis (p < 0.05) are fitted as directional vectors into the multidimensional space. Clusters are represented using colour code in the legend, and ellipses represent 95% confidence intervals of the clusters. (b) Barplot reports the coefficients of determination (R²) from the OTUs selected by the environmental fitting analysis (p < 0.05). Each bar is colored based on the best alignment between a significant OTU and a cluster using a cosine analysis. The asterisks highlight significance levels of each OTU (*, p<0.05; **, p<0.01; ***, p<0.001).

**Supplementary Table 5**. Table reports the results of the environmental fitting analysis depicted in Supplementary Figure S3. Table reports the significant OTUs together with the species-level taxonomic assignment, the related cluster calculated using cosine similarity and the output values from *envfit* analysis, i.e. coefficients of determination (R²) values and p-values. Significant effects are also highlighted using asterisks (*, p<0.05; **, p<0.01; ***, p<0.001).

| OTU | Species-level assignment | Cluster | R2 | p-value | Sign |
| --- | --- | --- | --- | --- | --- |
| RS_GCF_029024925.1 | *Enterococcus faecalis* | 3 | 0.9182 | 0.000999 | *** |
| GB_GCA_905197905.1 | *Sutterella sp905197905* | 1 | 0.6175 | 0.000999 | *** |
| RS_GCF_006742205.1 | *Staphylococcus epidermidis* | 4 | 0.2849 | 0.000999 | *** |
| GB_GCA_902469435.1 | *Collinsella sp902469435* | 5 | 0.1497 | 0.000999 | *** |
| GB_GCA_004555055.1 | *Alloprevotella sp004555055* | 1 | 0.1987 | 0.000999 | *** |
| GB_GCA_900764415.1 | *Collinsella sp900764415* | 1 | 0.2945 | 0.000999 | *** |
| RS_GCF_000194175.1 | *Escherichia coli F* | 1 | 0.1944 | 0.000999 | *** |
| RS_GCF_000026225.1 | *Escherichia fergusonii* | 1 | 0.1618 | 0.000999 | *** |
| RS_GCF_002901705.1 | *Staphylococcus lugdunensis* | 4 | 0.1069 | 0.000999 | *** |
| RS_GCF_000269965.1 | *Bifidobacterium infantis* | 5 | 0.0887 | 0.000999 | *** |
| RS_GCF_002931555.1 | *Pseudoclavibacter sp002931555* | 4 | 0.1173 | 0.000999 | *** |
| RS_GCF_000759775.1 | *Escherichia albertii* | 1 | 0.1279 | 0.000999 | *** |
| RS_GCF_002902725.1 | *Staphylococcus caprae* | 3 | 0.0976 | 0.001998 | ** |
| RS_GCF_900458815.1 | *Staphylococcus saccharolyticus* | 4 | 0.1285 | 0.001998 | ** |
| RS_GCF_004211955.1 | *Escherichia sp004211955* | 1 | 0.0695 | 0.004995 | ** |
| RS_GCF_014836715.1 | *Escherichia whittamii* | 1 | 0.0693 | 0.005994 | ** |
| RS_GCF_000175635.1 | *Corynebacterium tuberculostearicum C* | 4 | 0.0498 | 0.008991 | ** |
| RS_GCF_013408445.1 | *Corynebacterium tuberculostearicum* | 4 | 0.0511 | 0.008991 | ** |
| RS_GCF_030506365.1 | *Corynebacterium tuberculostearicum E* | 4 | 0.0519 | 0.008991 | ** |
| GB_GCA_023399815.1 | *Stenotrophomonas sp023399815* | 1 | 0.0564 | 0.020979 | * |
| RS_GCF_002901845.1 | *Staphylococcus hominis* | 4 | 0.0437 | 0.022977 | * |
| RS_GCF_002269255.1 | *Klebsiella quasivariicola* | 3 | 0.0406 | 0.043956 | * |

To assess similarity between microbial community clusters, hierarchical clustering was performed on a Bray-Curtis dissimilarity matrix. The Bray-Curtis distances were computed from normalised abundance (relative abundance) data using the *vegdist* function from the "vegan" package. Hierarchical clustering was then conducted using the *hclust* function with the average linkage method (UPGMA). To evaluate the fidelity of the dendrogram in representing the original dissimilarities, cophenetic distances were calculated. The correlation between the original Bray-Curtis distance matrix and the cophenetic distance matrix was computed using Pearson’s correlation. A correlation coefficient (r) greater than 0.75 (r = 0.83) was interpreted as indicating a good fit between the dendrogram structure and the original dissimilarities. The dendrogram structure (Supplementary Figure S5) was produced by using the "ggdendro" package v0.2.^7^

**Supplementary Figure 5.** A dendrogram represents hierarchical clustering analysis performed using the average linkage method and based on a Bray-Curtis dissimilarity matrix from normalised abundance. Tree branches were drawn as line segments, and sample nodes were displayed as coloured points, according to the assigned cluster.

*Taxonomic signature associated with the clusters*

To provide an overview of the taxonomic characterization of the identified clusters, the phylum and genus -level relative abundances per sample were computed. OTUs lacking phylum or genus annotations were excluded prior to downstream analyses. Samples were grouped according to the predefined clusters, and cluster-specific taxonomic abundances were calculated by summing OTU abundances within each taxon and cluster. Relative abundances were then obtained by normalizing each taxon’s abundance to the total abundance within the corresponding cluster. To focus the analysis on consistently represented and biologically relevant taxa while reducing noise from low-abundance features, taxa were filtered using combined global abundance and prevalence criteria. Phyla were retained if they accounted for at least 5% of the global relative abundance across the dataset and were detected in at least 0.5% of samples. Genera were filtered based on a global relative abundance of at least 10% and presence in at least 0.05% of samples. The selected taxa were subsequently summarized in a cluster-level Supplementary Table 6.

General taxonomic profiling showed cluster-specific variations in both phylum and genus levels (Supplementary Table S6). At the phylum level, Bacillota was most represented in clusters 3, 4, and 5, accounting for more than half of the total relative abundance in these clusters, while Actinomycetota was markedly represented in cluster 2. In contrast, Pseudomonadota was the most abundant phylum in cluster 1, then the phylum Bacteroidota contributed more modestly across all clusters. These phylum-level patterns were reflected by the genus-level profiling. Cluster 3 was strongly associated with *Enterococcus*, cluster 4 with *Staphylococcus*, and cluster 5 with *Streptococcus*. *Bifidobacterium* and *Collinsella* were mainly represented in clusters 2. In contrast, *Escherichia* and *Sutterella* were primarily associated with cluster 1, mirroring the dominance of Pseudomonadota in this cluster. Overall, the concordant stratification observed at both taxonomic levels highlights the presence of distinct microbial community structures across clusters.

**Supplementary Table 6**. Cluster-specific relative abundances of selected taxa. Rows correspond to phyla and genera retained after filtering based on combined global abundance and prevalence criteria (phyla: ≥ 5% global relative abundance and ≥ 0.5% sample prevalence; genera: ≥ 10% global relative abundance and ≥ 0.05% sample prevalence). Columns report taxonomy level and related taxa for each cluster (columns from 1 to 5). Values indicate the relative abundance of each phylum or genus within each cluster.

| Taxonomy-level | Taxa | 1 | 2 | 3 | 4 | 5 |
| --- | --- | --- | --- | --- | --- | --- |
| Phylum | Bacillota | 0.2083 | 0.1043 | 0.7412 | 0.5296 | 0.5196 |
|  | Actinomycetota | 0.1416 | 0.5683 | 0.0794 | 0.2908 | 0.3182 |
|  | Pseudomonadota | 0.4986 | 0.1900 | 0.1010 | 0.0459 | 0.0696 |
|  | Bacteroidota | 0.1159 | 0.0596 | 0.0222 | 0.0285 | 0.0566 |
| Genus | Enterococcus | 0.105 | 0.024 | 0.611 | 0.021 | 0.048 |
|  | Bifidobacterium | 0.045 | 0.219 | 0.049 | 0.071 | 0.269 |
|  | Staphylococcus | 0.015 | 0.032 | 0.060 | 0.476 | 0.003 |
|  | Streptococcus | 0.043 | 0.019 | 0.036 | 0.009 | 0.416 |
|  | Collinsella | 0.092 | 0.345 | 0.023 | 0.013 | 0.043 |
|  | Escherichia | 0.167 | 0.129 | 0.034 | 0.005 | 0.038 |
|  | Sutterella | 0.277 | 0.040 | 0.015 | 0.002 | 0.021 |

To identify differentially abundant operational taxonomic units (OTUs) among clusters, the OTUs present in at least 10% of the samples were filtered to avoid bias related to rare OTUs, then transformed into relative abundance percentages. Differences among clusters were inspected by using the Kruskal-Wallis rank sum test, adjusting the p-value using the false discovery rate (FDR) method. OTUs with FDR-adjusted p-values < 0.05 were considered significantly different across clusters (Supplementary Figure S6) and retained for post hoc pairwise testing. For each significant OTU, a pairwise Wilcoxon rank-sum test (FDR correction) was performed between clusters (Supplementary Table S7). Significantly different OTUs were further explored through heatmap analysis of percentage abundance. Heatmaps generated with the Significant OTUs were displayed using hierarchical clustering analysis produced using the package "ComplexHeatmap" v2.18,^8,^ applying Euclidean distance and hierarchical clustering with the Ward D2 method (Figure 3d).

**Supplementary Figure 6.** Different OTUs abundances among clusters. Bar plots represent the mean OTU relative abundance percentages and standard error across clusters (colour scheme). The OTUs are selected by using the Kruskal-Wallis test (p < 0.05), then with the Wilcoxon rank test (FDR correction method). For each OTU, the corresponding species-level taxonomic assignment is reported.

**Supplementary Table 7.** The table reports the differences in OTUs' relative abundances among clusters assessed by using the Wilcoxon rank sum test. The p-values are adjusted by using the FDR correction method. The table reports the OTUs' annotations and related species-level taxonomic assignment. Significant comparisons are highlighted using asterisks (*, p<0.05; **, p<0.01; ***, p<0.001).

*Inferential analysis between maternal lifestyle parameters and clusters*

Maternal dietary, lifestyle-related variables and gestational age in weeks were analyzed in relation to previously defined clusters. Univariate differences among clusters for each variable were assessed using Kruskal-Wallis test, highlighting the absence of significant differences among clusters observed for any of the individual variables. In detail, gestational age in weeks (GA), weight gain (WG), end-pregnancy weight (EPW), end-pregnancy body mass index (EPBMI), food frequency questionnaire score (FFQ), dietary habits score (DH), physical activity score (PA), and Mediterranean Lite adherence score (MLS) showed p-values > 0.179, indicating comparable distributions across clusters for each variable considered independently. For graphical comparison of multivariate profiles among clusters, all variables were first normalized using min-max scaling to a common 0-1 range, preserving relative differences while ensuring comparability across different measurement scales and represented using radar-plot layout in Figure 3g. To assess overall multivariate differences among clusters while jointly considering all seven variables, a permutational multivariate analysis of variance (adonis PERMANOVA) was performed using the *adonis2* function of "vegan" package v2.6.10. Prior to analysis, variables were standardized using z-score transformation to reduce biases related to different scales. Euclidean distances were computed from the standardized matrix, and adonis PERMANOVA was conducted with 1’000 permutations. Multivariate patterns were further explored using PCoA based on the same euclidean distance matrix used for multivariate analysis.

The multivariate comparison of clusters revealed no significant overall differences in the joint distribution of the eight variables (R² = 0.027, p = 0.672), in accordance to the PCoA which did not show discrete separation among clusters (Figure 3f), indicating substantial overlap among clusters in the multidimensional space defined by the analyzed variables.

The association between clusters and children's biometric parameters (weight, height, and head circumference at birth) was also tested using univariate analysis as described above, but no significant differences were observed (child weight: p = 0.26; child length: p = 0.24; head circumference: p = 0.22).

*Predicted functional profiling using PICRUSt2*

A small number of input genomes (GB_GCA_024160165.1, RS_GCF_000158195.2, GB_GCA_023399815.1, RS_GCF_000154445.1) were excluded due to insufficient alignment to the reference phylogeny (minimum alignment proportion < 0.8), as recommended by PICRUSt2, ensuring conservative and reliable functional predictions. All samples exhibited weighted Nearest Sequenced Taxon Index (NSTI) values below the recommended threshold (2.0), indicating high prediction reliability and no quality-related bias.

*Multivariable modelling of predicted bacterial functions and bacterial OTUs*

Multivariable associations between predicted microbial functions and host clinical variables were assessed using MaAsLin2 v1.16^9^, fitting an independent linear model for each bacterial feature (KO, EC number, MetaCyc pathway or microbial genome). In each model, feature abundance was treated as the dependent variable, whereas gestational age, GWG, maternal age and pre-pregnancy BMI were included simultaneously as fixed effects. GWG was treated as a categorical variable using the "adequate" group as the reference category. Because PICRUSt2 outputs represent copy number-corrected predicted functional abundances, no additional normalization was applied to KEGG Orthologs (KOs), EC numbers (EC) or MetaCyc pathways abundance tables. Low-prevalence functional features were removed independently from each functional dataset, thus KOs, EC and MetaCyc pathways detected in fewer than 5% of samples were excluded from the analyses in order to reduce the influence of sparse features and improve model stability. Functional abundances were log-transformed using the default MaAsLin2 logarithmic transformation, and associations were estimated using multivariable linear regression.

Although MaAsLin2 reports associations using a default false discovery rate (FDR) threshold of q < 0.25 as an exploratory criterion, a more conservative significance threshold of q < 0.05 was adopted in the present study to minimize false-positive discoveries and to increase confidence in the reported associations. Anyway, associations with q-values between 0.05 and 0.25 were reported (grey points in Figure S7) for completeness but were not considered significant. To assess biological interpretation, significant KEGG Orthologs and Enzyme Commission (EC) numbers were annotated using the KEGG REST Application Programming Interface (API) implemented in the “KEGGREST” package v1.42^10^, while MetaCyc pathway identifiers were verified using the MetaCyc database (https://metacyc.org/).

No significant associations were observed between predicted microbial functions and GWG, maternal age or pre-pregnancy BMI after multivariable adjustment. Conversely, gestational age showed significant associations across all three levels of functional annotation. All the significant (q < 0.05) features identified (9 EC numbers, 21 KEGG Orthologs and 1 MetaCyc pathway) were negatively associated with gestational age, indicating a progressive decrease in their predicted abundance with increasing gestational age (red points in Figure S7 and Table S8).

Significant KO were primarily associated with functions involved in sulfur metabolism and redox reactions, including adenylylsulfate reduction (K00395) and rubredoxin-dependent electron transfer (K05297), together with functions related to amino acid and carbohydrate metabolism (K02203, K00132, K13810), lipopolysaccharides biosynthesis (K12992), bacterial stress response and survival (K06298, K07216, K19165), and other cellular processes including nucleoside metabolism (K06928) and polyamine metabolism (K12256). Most significant KOs were detected in fewer than 20 subjects, indicating that these associations were driven by relatively low-prevalence predicted functions within the study population.

Consistent with these findings, the significantly associated EC numbers mainly represented enzymes involved in sulfur metabolism and oxidoreductase activities, including adenylylsulfate reductase (EC 1.8.99.2), rubredoxin-NAD⁺ reductase (EC 1.18.1.1) and heterodisulfide reductase-associated enzymes (EC 1.8.7.3, EC 1.8.98.4, EC 1.8.98.5 and EC 1.8.98.6), together with enzymes involved in nucleotide metabolism (EC 3.6.1.15), amino acid metabolism (EC 1.3.99.33) and polyamine metabolism (EC 2.6.1.113).

A single MetaCyc pathway, P221-PWY (octane oxidation), showed a significant negative association with gestational age. This predicted pathway was detected in only 16 samples (12.1% of subjects within the dataset tested), suggesting that although the statistical association was robust, its contribution to the overall functional landscape was restricted to a relatively small subset of individuals.

Although the associations between gestational age and KOs, EC numbers and MetaCyc pathway were statistically robust after multiple-testing correction (q < 0.05), the corresponding functional features were detected in a relatively small proportion of the study population. Significant KOs and EC numbers were detected in a range of 7-22 of 132 individuals corresponding to 5.3-16.7% of subjects within the dataset, and the only significant MetaCyc pathway, P221-PWY, was detected in 16 samples (12.1%). Therefore, these associations likely reflect functional signatures confined to a part of the microbial communities represented in the cohort rather than widespread functional characteristics of the entire population.

**Supplementary Figure 7**. Effect size plot showing the multivariable associations between predicted bacterial functional features and gestational age identified using MaAsLin2. Predicted functional profiles inferred by PICRUSt2 were separately inferred at three hierarchical levels, including Enzyme Commission (EC) numbers, KEGG Orthologs (KOs) and MetaCyc pathways (PWYs). Points represent regression coefficients estimated from multivariable linear models while horizontal bars indicate standard errors. Negative coefficients indicate a decrease in predicted feature abundance with increase of gestational age. Features are coloured according to statistical significance after false discovery rate correction (q < 0.05, red points) or default MaAsLin2 significance threshold (0.05 ≤ q < 0.25, grey points). Labels include the number of samples in which each feature was detected (n). Only features retained after prevalence filtering (detected in ≥5% of samples) are reported. All associations reaching the significance threshold (q < 0.05) are observed exclusively for gestational age.

**Supplementary Table 8**. Significant predicted bacterial functional features associated with gestational age identified by multivariable association analysis MaAsLin2. Functional features were inferred from PICRUSt2 predictions and analysed using MaAsLin2 and the table reports all significantly associated Enzyme Commission (EC) numbers, KEGG Orthologs (KOs) and MetaCyc pathway (PWY) identified using a FDR threshold of q < 0.05. Functional annotations for each EC number, KO and pathway were reported. Prevalence indicates the number of samples in which each predicted functional feature was detected within the dataset. Multiple annotations (Identifier) are reported for the same EC number when alternative enzyme names or gene names are available in the KEGG database.

| **Level** | **Identifier** | **Functional annotation** | **q-value** | **Prevalence** |
| --- | --- | --- | --- | --- |
| EC | 1.8.7.3 | ferredoxin:CoB-CoM heterodisulfide reductase | 1.18E-05 | 8 |
| EC | 1.8.7.3 | hdrABC (gene names) | 1.18E-05 | 8 |
| EC | 1.8.7.3 | hdrA1B1C1 (gene names) | 1.18E-05 | 8 |
| EC | 1.8.7.3 | hdrA2B2C2 (gene names) | 1.18E-05 | 8 |
| EC | 1.8.98.4 | coenzyme F420:CoB-CoM  heterodisulfide,ferredoxin reductase | 1.18E-05 | 8 |
| EC | 1.8.98.4 | hdrA2B2C2 (gene names) | 1.18E-05 | 8 |
| EC | 1.8.98.6 | formate:CoB-CoM heterodisulfide,ferredoxin reductase | 1.18E-05 | 8 |
| EC | 1.8.98.5 | H2:CoB-CoM heterodisulfide,ferredoxin reductase | 1.68E-05 | 8 |
| EC | 1.18.1.1 | rubredoxin---NAD+ reductase | 2.11E-04 | 15 |
| EC | 1.18.1.1 | rubredoxin reductase | 2.11E-04 | 15 |
| EC | 1.18.1.1 | rubredoxin-nicotinamide adenine dinucleotide reductase | 2.11E-04 | 15 |
| EC | 1.18.1.1 | dihydronicotinamide adenine  dinucleotide-rubredoxin reductase | 2.11E-04 | 15 |
| EC | 1.18.1.1 | reduced nicotinamide adenine  dinucleotide-rubredoxin reductase | 2.11E-04 | 15 |
| EC | 1.18.1.1 | NADH-rubredoxin reductase | 2.11E-04 | 15 |
| EC | 1.18.1.1 | rubredoxin-NAD reductase | 2.11E-04 | 15 |
| EC | 1.18.1.1 | NADH: rubredoxin oxidoreductase | 2.11E-04 | 15 |
| EC | 1.18.1.1 | DPNH-rubredoxin reductase | 2.11E-04 | 15 |
| EC | 1.18.1.1 | NADH-rubredoxin oxidoreductase | 2.11E-04 | 15 |
| EC | 1.8.99.2 | adenylyl-sulfate reductase | 3.11E-04 | 17 |
| EC | 1.8.99.2 | adenosine phosphosulfate reductase | 3.11E-04 | 17 |
| EC | 1.8.99.2 | adenosine 5'-phosphosulfate reductase | 3.11E-04 | 17 |
| EC | 1.8.99.2 | APS-reductase | 3.11E-04 | 17 |
| EC | 1.8.99.2 | APS reductase | 3.11E-04 | 17 |
| EC | 1.8.99.2 | AMP, sulfite:(acceptor)  oxidoreductase (adenosine-5'-phosphosulfate-forming) | 3.11E-04 | 17 |
| EC | 3.6.1.15 | nucleoside-triphosphate phosphatase | 5.48E-04 | 10 |
| EC | 3.6.1.15 | Nucleoside-triphosphatase | 5.48E-04 | 10 |
| EC | 3.6.1.15 | nucleoside triphosphate phosphohydrolase | 5.48E-04 | 10 |
| EC | 3.6.1.15 | nucleoside-5-triphosphate phosphohydrolase | 5.48E-04 | 10 |
| EC | 3.6.1.15 | nucleoside 5-triphosphatase | 5.48E-04 | 10 |
| EC | 3.6.1.15 | unspecific diphosphate phosphohydrolase | 5.48E-04 | 10 |
| EC | 1.3.99.33 | urocanate reductase | 0.0026 | 8 |
| EC | 1.3.99.33 | urdA (gene name) | 0.0026 | 8 |
| EC | 2.6.1.113 | putrescine---pyruvate transaminase | 0.0353 | 8 |
| EC | 2.6.1.113 | spuC (gene name) | 0.0353 | 8 |
| KO | K13810 | transaldolase / glucose-6-phosphate isomerase  [EC:2.2.1.2 5.3.1.9] | 5.24E-09 | 10 |
| KO | K03388 | heterodisulfide reductase subunit A2  [EC:1.8.7.3 1.8.98.4 1.8.98.5 1.8.98.6] | 1.76E-05 | 7 |
| KO | K03389 | heterodisulfide reductase subunit B2  [EC:1.8.7.3 1.8.98.4 1.8.98.5 1.8.98.6] | 2.41E-05 | 8 |
| KO | K02203 | phosphoserine / homoserine phosphotransferase  [EC:3.1.3.3 2.7.1.39] | 5.50E-05 | 15 |
| KO | K00395 | adenylylsulfate reductase, subunit B [EC:1.8.99.2] | 6.19E-05 | 14 |
| KO | K06298 | germination protein M | 3.52E-04 | 18 |
| KO | K05297 | rubredoxin---NAD+ reductase [EC:1.18.1.1] | 4.47E-04 | 15 |
| KO | K07576 | metallo-beta-lactamase family protein | 6.15E-04 | 18 |
| KO | K07216 | hemerythrin | 0.0012 | 17 |
| KO | K06928 | nucleoside-triphosphatase [EC:3.6.1.15] | 0.0012 | 10 |
| KO | K12240 | L-cysteine---[L-cysteinyl-carrier protein]  ligase PchF [EC:6.2.1.69] | 0.0026 | 11 |
| KO | K13730 | internalin A | 0.0034 | 14 |
| KO | K11184 | catabolite repression HPr-like protein | 0.0042 | 7 |
| KO | K17363 | urocanate reductase [EC:1.3.99.33] | 0.0042 | 8 |
| KO | K05303 | O-methyltransferase [EC:2.1.1.-] | 0.0042 | 7 |
| KO | K19872 | fukutin [EC:2.7.8.-] | 0.0100 | 11 |
| KO | K12992 | O-antigen biosynthesis  alpha-1,3-rhamnosyltransferase [EC:2.4.1.377] | 0.0110 | 22 |
| KO | K04784 | yersiniabactin nonribosomal peptide synthetase | 0.0110 | 12 |
| KO | K19165 | antitoxin Phd | 0.0110 | 13 |
| KO | K00132 | acetaldehyde dehydrogenase (acetylating) [EC:1.2.1.10] | 0.0227 | 22 |
| KO | K12256 | putrescine---pyruvate transaminase [EC:2.6.1.113] | 0.0448 | 8 |
| PWY | P221.PWY | octane oxidation pathway | 0.0001 | 16 |

To determine whether the functional associations identified by PICRUSt2 were accompanied by changes in the observed OTUs composition, multivariable association analysis using MaAsLin2 v1.16.0 was performed directly on the OTU-level abundance table. OTUs detected in fewer than 5% of samples were excluded prior to analysis to reduce sparsity. The OTUs abundances were normalized using total sum scaling (TSS) and log-transformed using the default MaAsLin2 transformation. Independent multivariable linear models were fitted using MaAsLin2 v1.16.0, including gestational age, GWG, maternal age and pre-pregnancy BMI as fixed effects, with the adequate GWG category used as the reference level. Statistical significance was defined as *q* < 0.05 after false discovery rate correction.

Only one OTU was significantly associated with the gestational age after multivariable adjustment and false discovery rate correction (q < 0.05). Specifically, the predicted abundance of RS_GCF_002269255.1 (*Klebsiella quasivariicola*) showed a significant negative association with gestational age (coefficient = -0.459, se = 0.116, q = 0.043), indicating a progressive decrease in abundance with increasing gestational age. No significant associations were identified for GWG, maternal age or pre-pregnancy BMI. The significant OTU was detected in only 7 of the 132 study participants (5.3%), suggesting that this taxonomic association was confined to a relatively small subset of the cohort.

*Unsupervised functional profiling of cluster-associated bacterial OTUs*

Using the PICRUSt2 pipeline, predicted functional profiles were inferred from the bacterial OTUs identified in the dataset. Predicted KO abundances were generated and used for downstream functional analyses. In parallel, PICRUSt2-generated OTU-level pathway predictions were integrated with the corresponding predicted OTU abundances to estimate weighted pathway abundances for each sample. Specifically, pathway abundances were calculated by weighting the predicted abundance of each OTU according to its predicted genomic capacity to encode individual metabolic pathways, thereby generating sample-level estimates of the functional potential of the bacterial community.

Then, to investigate the predicted functional potential associated with specific OTU clusters, the set of 21 reference OTU in which their abundance significantly changed among clusters (OTUs represented in Supplementary Figure S6) was considered. For each target OTU, pathways with non-zero predicted contributions were extracted and compiled into a binary pathway-by-OTU matrix. OTU-pathway intersections were represented using an "UpSet" plot, generated using the "UpSetR" package v1.4.0^11^ , enabled identification of "core pathways" (present in all OTUs), "accessory pathways" (intended as OTUs shared by heterogeneous subsets of OTUs), and "unique pathways" (specific to a single OTU) (Figure S8).

**Supplementary Figure 8** . UpSet plot of predicted metabolic pathway intersections from PICRUSt2 pipeline across 21 target OTUs. The UpSet plot illustrates the distribution of predicted pathways among selected microbial OTUs. Vertical bars represent the number of pathways shared by specific genome combinations, while connected dots indicate participating OTUs.

All figures were produced using the graphics package "ggplot2" v3.5.2^12^ ^,^ and where necessary, the graphic rendering was improved using the open source software Inkscape (https://inkscape.org/).

**References of supplementary materials**

1. Pagès H, Aboyoun P, Gentleman R, DebRoy S (2024). Biostrings: Efficient manipulation of biological strings. R package version 2.70.3, <https://bioconductor.org/packages/Biostrings>.
2. Andersen KS, Kirkegaard RH, Karst SM, Albertsen M (2018). ampvis2: an R package to analyse and visualise 16S rRNA amplicon data. bioRxiv. doi: <https://doi.org/10.1101/299537>.
3. Turunen J, Tejesvi MV, Paalanne N, et al. (2024). Investigating prenatal and perinatal factors on meconium microbiota: a systematic review and cohort study. *Pediatr Res* 95:135–145. <https://doi.org/10.1038/s41390-023-02783-z>.
4. Oksanen J, Simpson G, Blanchet F, et al. (2025). vegan: Community Ecology Package. R package version 2.6-10, [https://CRAN.R-project.org/package=vegan](https://cran.r-project.org/package=vegan).
5. Fox J, Weisberg S (2019). *An R Companion to Applied Regression*, Third edition. Sage, Thousand Oaks CA. <https://www.john-fox.ca/Companion/>.
6. Maechler M, Rousseeuw P, Struyf A, et al. (2022). cluster: Cluster Analysis Basics and Extensions. R package version 2.1.4.
7. De Vries A, Ripley BD (2024). ggdendro: Create Dendrograms and Tree Diagrams Using 'ggplot2'. R package version 0.2.0, [https://CRAN.R-project.org/package=ggdendro](https://cran.r-project.org/package=ggdendro).
8. Gu Z, Eils R, Schlesner M (2016). Complex heatmaps reveal patterns and correlations in multidimensional genomic data. *Bioinformatics* 32(18):2847–2849.
9. Mallick H, Rahnavard A, McIver LJ, Ma S, Zhang Y, et al. (2021). Multivariable association discovery in population-scale meta-omics studies. *PLoS computational biology*, *17*(11), e1009442.
10. Tenenbaum, D., & Maintainer, B. P. (2023). *KEGGREST: Client-side REST access to the Kyoto Encyclopedia of Genes and Genomes (KEGG). R package version 1.42.0. Bioconductor*.
11. Gehlenborg N (2019). UpSetR: A More Scalable Alternative to Venn and Euler Diagrams for Visualizing Intersecting Sets. R package version 1.4.0, [https://CRAN.R-project.org/package=UpSetR](https://cran.r-project.org/package=UpSetR).
12. Wickham H (2016). *ggplot2: Elegant Graphics for Data Analysis*. Springer-Verlag New York.
